# Regulation of volume-regulated anion channels alters sensitivity to platinum chemotherapy

**DOI:** 10.1126/sciadv.adr9364

**Published:** 2024-12-13

**Authors:** Lily Elizabeth R. Feldman, Saswat Mohapatra, Robert T. Jones, Mathijs Scholtes, Charlene B. Tilton, Michael V. Orman, Molishree Joshi, Cailin S. Deiter, Travis P. Broneske, Fangyuan Qu, Corazon Gutierrez, Huihui Ye, Eric T. Clambey, Sarah Parker, Tokameh Mahmoudi, Tahlita Zuiverloon, James C. Costello, Dan Theodorescu

**Affiliations:** ^1^ Department of Pharmacology, University of Colorado Anschutz Medical Campus, Aurora, CO, USA.; ^2^ Cedars-Sinai Samuel Oschin Comprehensive Cancer Institute, Los Angeles, CA, USA.; ^3^ Department of Urology, Erasmus MC Cancer Institute, Erasmus University Medical Center, Rotterdam, Netherlands.; ^4^ Functional Genomics Facility, University of Colorado Anschutz Medical Campus, Aurora, CO, USA.; ^5^ Department of Pathology and Laboratory Medicine, Cedars-Sinai Medical Center, Los Angeles, CA, USA.; ^6^ Department of Anesthesiology, University of Colorado Anschutz Medical Campus, Aurora, CO, USA.; ^7^ Smidt Heart Institute & Advanced Clinical Biosystems Research Institute, Cedars Sinai Medical Center, Los Angeles, CA, USA.; ^8^ Department of Biochemistry, Erasmus University Medical Center, Rotterdam, Netherlands.; ^9^ Department of Pathology, Erasmus University Medical Center, Rotterdam, Netherlands.; ^10^ University of Colorado Comprehensive Cancer Center, University of Colorado Anschutz Medical Campus, Aurora, CO, USA.; ^11^ Department of Urology, Cedars-Sinai Medical Center, Los Angeles, CA, USA.

## Abstract

Cisplatin-based chemotherapy is used across many common tumor types, but resistance reduces the likelihood of long-term survival. We previously found the puromycin-sensitive aminopeptidase, NPEPPS, as a druggable driver of cisplatin resistance in vitro and in vivo and in patient-derived organoids. Here, we present a general mechanism where NPEPPS interacts with the volume-regulated anion channels (VRACs) to control cisplatin import into cells and thus regulate cisplatin response across a range of cancer types. We also find the NPEPPS/VRAC gene expression ratio is a predictive measure of cisplatin response in multiple cancer cohorts, showing the broad applicability of this mechanism. Our work describes a specific mechanism of cisplatin resistance, which, given the characteristics of NPEPPS as a drug target, has the potential to improve cancer patient outcomes. In addition, we describe an intracellular mechanism regulating VRAC activity, which is critical for volume regulation in normal cells – a finding with functional implications beyond cancer.

## Teaser

A general mechanism that controls platinum drug import can be targeted to improve chemotherapy response in patients with cancer.

## INTRODUCTION

Cisplatin is a widely used chemotherapeutic and first-line therapy for many cancer types, including bladder, ovarian, cervical, testicular, lung, breast, sarcomas, lymphomas, and leukemias (*1*, *2*). Platinum family drugs are present in the regimens of nearly 50% of all patients who receive chemotherapy (*3*, *4*). Furthermore, in advanced bladder cancer (BCa), it has been shown that combination therapy with checkpoint inhibitors resulted in substantially better outcomes (*5*). Cisplatin’s primary mechanism of action is DNA damage, but tumor cells use many resistance mechanisms to evade treatment, leading to poor outcomes for patients (*6*–*9*). These resistance mechanisms present previously unexplored opportunities to improve patient survival and lower the effective platinum dosage to mitigate side effects and toxicities (*10*, *11*).

We recently found that puromycin-sensitive aminopeptidase, NPEPPS, is up-regulated in the context of platinum resistance, resulting in decreased platinum accumulation in BCa cells (*12*). This is a previously unidentified action of NPEPPS, independent of its putative function to digest peptide fragments into amino acids via cleavage of glutamine-glutamine bonds (*13*). We also showed that genetic and therapeutic inhibition of NPEPPS increased sensitivity to cisplatin in cell culture, xenograft models, and ex vivo patient-derived organoids (*12*). We lastly showed that NPEPPS is therapeutically actionable, making it a promising target for drug development. However, the mechanism by which NPEPPS regulates intracellular platinum drug concentrations and subsequent response was not determined.

The volume-regulated anion channel (VRAC) was found to regulate cell volume in response to osmotic stress (*14*). VRAC subunit proteins (LRRC8A-E) are composed of an extracellular loop, seven transmembrane α helices, and the intracellular leucine-rich repeat (LRR) domains for which they are named (*15*). VRAC pores are formed when six subunits, including LRRC8A and at least one other subunit, assemble at the cell membrane, where stimuli on the cytosolic LRR domains are known to influence channel activity (*16*, *17*). In general, VRACs support volume homeostasis by transporting anions across the cell membrane to trigger or protect against the apoptotic volume decrease cascade that precedes apoptosis (*18*–*20*). VRAC activity can be stimulated with hypotonic solutions, platinum drugs, and reactive oxygen species (*17*, *21*). VRACs transport many substrates, with transport activity depending on the subunit composition of the channel and the cell type expressing it. The VRACs have been reported to import up to 70% of intracellular cisplatin and carboplatin along with transporting taurine, iodide, and chloride anions (*22*). Studies in astrocytes have shown that VRACs can transport neurotransmitters such as γ-aminobutyric acid and glutamate across the cell membrane (*23*–*26*). Results in pancreatic islet β cells, smooth muscle cells, and adipocytes suggest that VRACs can respond to the presence of glucose to influence insulin signaling and glucose tolerance (*27*–*30*). LRRC8A and LRRC8D have emerged as vital subunits for functional VRAC assembly and activity, particularly related to platinum drug import; the contributions of LRRC8B, LRRC8C, and LRRC8E remain unresolved.

The many substrates transported by the VRACs highlight its importance across many areas of research, but we lack a complete understanding of what controls this crucial transporter. Deneka *et al.* generated synthetic nanobodies—small, antibody-like proteins—that bind to the intracellular LRR domain of VRAC subunits LRRC8A-LRRC8E, and found that these regulate channel activity, demonstrating that VRACs can be controlled through cytosolic protein-protein interactions (*16*). To our knowledge, no native, intracellular regulator of the VRAC has been identified in any system (*16*, *20*, *21*, *31*). Identification of such regulators may allow therapeutic enhancement of this channel’s platinum import function to improve cisplatin activity and patient survival across many cancer types. Furthermore, VRACs are essential to fundamental normal biological processes in human cells, and understanding how they are regulated by the cell itself has broad implications beyond platinum drug import and cancer therapeutics (*32*).

Here, we present the description of the molecular mechanisms underpinning NPEPPS’s role in driving resistance to cisplatin. We show that NPEPPS forms a complex with LRRC8A, an essential subunit of the VRAC, at the plasma membrane to block VRAC transport activity. We demonstrate that the NPEPPS-LRRC8A interaction leads to reduced cisplatin uptake in multiple types of human immortalized tumor and nontumor cell lines. We further show that this protein-protein interaction is susceptible to pharmacologic and genetic manipulation and that its disruption can increase platinum accumulation and drug sensitivity in cancer cells.

## RESULTS

### NPEPPS protein forms complexes with VRAC subunits

**Fig. 1.**
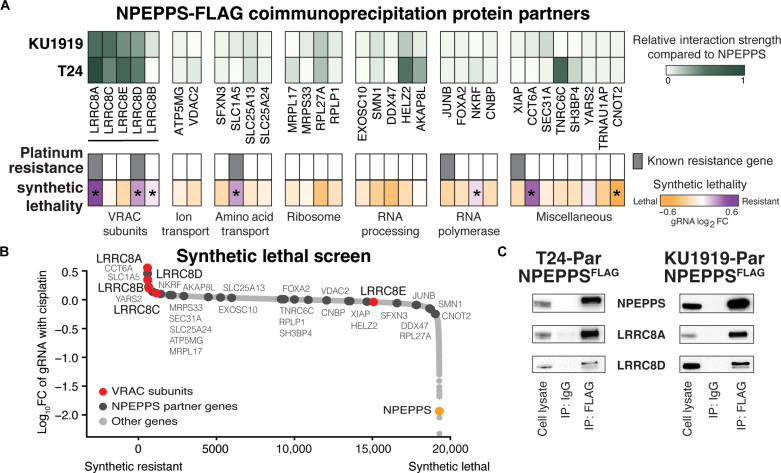
NPEPPS protein interaction partners. (**A**) The heatmap represents LC-MS/MS analysis of NPEPPS^FLAG^ pull-down using protein lysates from BCa cell lines (KU1919 and T24; *n* = 3 independent lysates per cell line). The relative strength of interaction is scaled from 0 to 1. Genes annotated as playing a role in platinum drug resistance by Huang *et al.* (*34*) are highlighted. Results from a CRISPR screen reported in Jones *et al.* (*12*) identifying synthetic gene-to-drug interactions against cisplatin-based chemotherapy in treatment-resistant cell lines are shown. *FDR < 0.05. (**B**) Proteins identified in the NPEPPS^FLAG^ screen as black dots and the VRAC subunits (LRRC8A-E) as red dots are mapped onto the CRISPR screen results. NPEPPS is highlighted in orange. (**C**) Affinity tag (FLAG), or IgG control, IP of protein lysates from NPEPPS^FLAG^ in KU1919 and T24 cells was immunoblotted for NPEPPS, LRRC8A, and LRRC8D.

We recently showed that NPEPPS regulates intracellular levels of cisplatin in human BCa (*12*). To identify the mechanism by which NPEPPS carries this out, we characterized the interaction partners of NPEPPS via immunoprecipitation (IP) followed by liquid chromatography–tandem mass spectrometry (LC-MS/MS). We overexpressed FLAG-tagged NPEPPS in KU1919 and T24 human BCa cells, selected to represent diverse patient backgrounds (KU1919 origin: 76-year-old male, East Asian genetic ancestry, intact TP53; T24 origin: 82-year-old female, European genetic ancestry, nonsense TP53 mutation) (*33*). We validated that NPEPPS^FLAG^ was expressed at the correct molecular weight and recognized by the NPEPPS antibody by anti-FLAG IP and Western blot (fig. S1). We performed IP targeting NPEPPS^FLAG^ followed by LC-MS/MS to identify 185 (KU1919) and 166 (T24) proteins enriched over non-FLAG controls [interaction intensity ≥ 1 and log_2_ fold change (FC) ≥ 1]. Intersecting these two lists, we identified 32 proteins in both cell lines (Fig. 1A, fig. S2, and table S1).

We prioritized the list of 32 proteins for their relationship to NPEPPS and relevance to cisplatin response (Fig. 1A) as follows. First, we used a database of genes involved in platinum response (*34*) to annotate interactions with previously reported platinum resistance–associated genes. Five NPEPPS interaction partners were identified, among which *LRRC8A* and *LRRC8D* stood out as the top genes with a known relationship to platinum resistance (*34*). Second, we leveraged our synthetic drug–gene lethality CRISPR screen data, representing a list of nearly 20,000 genes prioritized for their ability to alter sensitivity to cisplatin (*12*). The aggregate results across five human BCa cell lines revealed six genes that, when lost, made the cells resistant to cisplatin-based treatment [false discovery rate (FDR) < 0.05]. The intersection of these six genes and those known to drive platinum resistance narrowed the list to *LRRC8A*, *LRRC8D*, and solute carrier family 1 member 5 (*SLC1A5*).

To further prioritize our LC-MS/MS results, we mined the BioPlex interactome database (*35*) and found that NPEPPS interacts with the VRAC subunits but not SLC1A5 (fig. S3). In addition, VRAC subunits LRRC8A and LRRC8D, which have been shown to directly import cisplatin into cells (*22*, *36*, *37*), were the 1st and 11th most synthetic-resistant genes from our previous screen (Fig. 1B) and induced cisplatin resistance consistently across all five BCa cell lines (*12*). Given these results, we prioritized LRRC8A and LRRC8D in our target list and confirmed that both proteins were pulled down with NPEPPS^FLAG^ by coimmunoprecipitation (co-IP) in nonresistant parental T24 and KU1919 cells (Fig. 1C and fig. S4). We repeated the IP–LC-MS/MS to validate these findings using a native anti-NPEPPS antibody for IP and found that LRRC8A was consistently identified among the top hits in NPEPPS^WT^ KU1919 and T24 cells (fig. S5).

### NPEPPS mediates cisplatin import through the VRACs

**Fig. 2.**
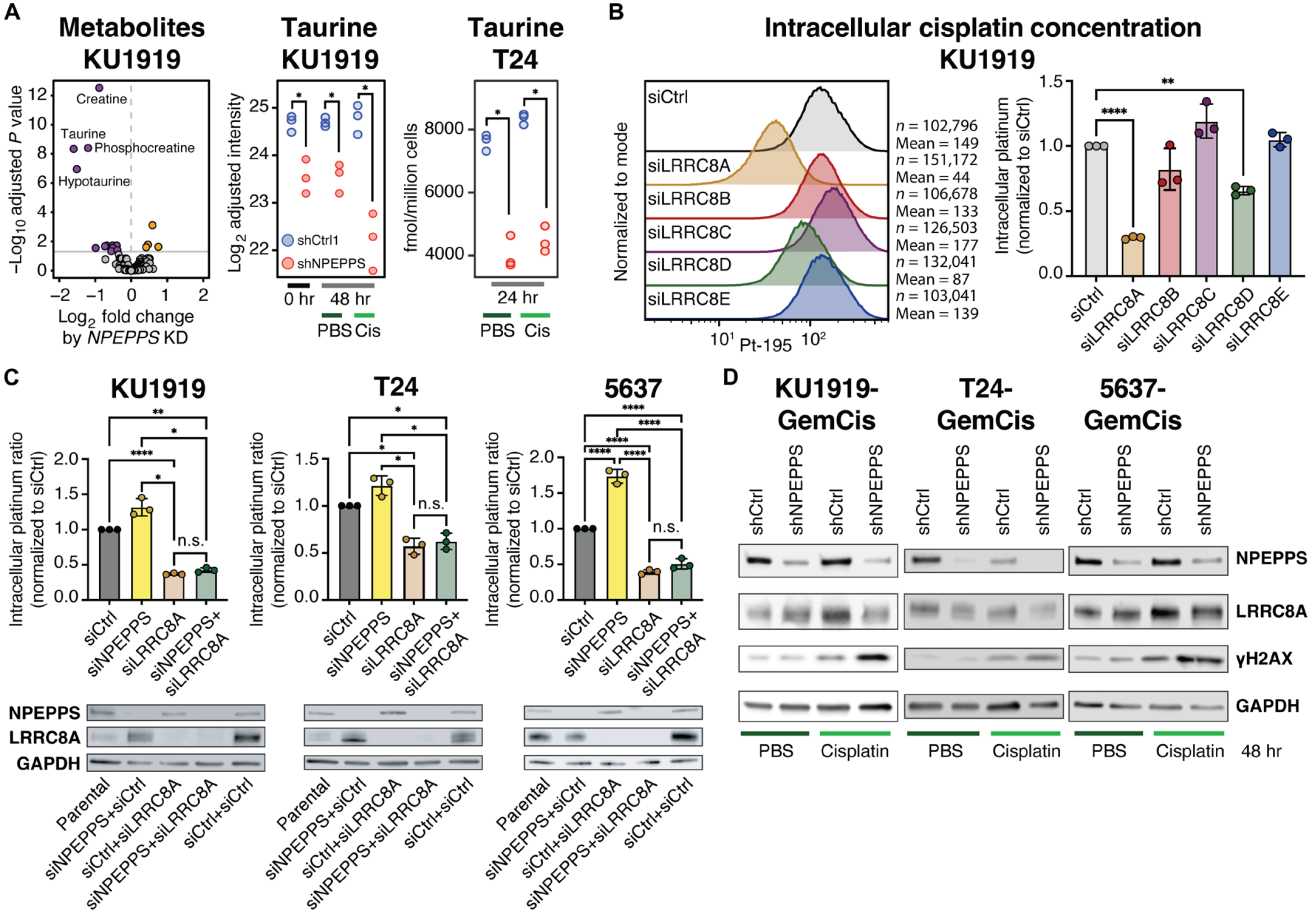
NPEPPS alters platinum import and DNA damage by modulating VRACs. (**A**) Untargeted metabolomics in KU1919 cells with shRNA-mediated NPEPPS suppression or control shRNA. Taurine levels are reported in control (PBS) and cisplatin (Cis) treatment conditions. Targeted metabolomic measured levels of taurine are reported in T24 cells in control (PBS) and cisplatin (Cis) treatment conditions. hr, hours. (**B**) CyTOF in KU1919 cells shows intracellular cisplatin levels after 4 hours of 10 μM cisplatin with siRNA-mediated suppression of VRAC subunits LRRC8A-E compared to control (scramble) siRNA. Median intracellular cisplatin measurements across biological triplicates were normalized to the siRNA control and compared using a one-way ANOVA (**P < 0.01; ****P < 0.001). (**C**) Intracellular cisplatin levels for KU1919, T24, and 5637 cells with siRNA-mediated knockdown of NPEPPS alone, LRRC8A alone, or the combination of NPEPPS and LRRC8A knockdown. All samples were normalized to siRNA control samples and compared using a one-way ANOVA (*P < 0.05; **P < 0.01; ****P < 0.0001). n.s., not significant. Immunoblot validation of the knockdowns is reported with native NPEPPS and LRRC8A antibodies. (**D**) KU1919, 5637, and T24 cells made resistant to GemCis were treated with cisplatin (10 μM) or PBS for 48 hours. Immunoblots with NPEPPS, LRRC8A, and phospho-γ-H2AX antibodies are shown, comparing shRNA-mediated knockdown of NPEPPS to shRNA scramble controls.

In addition to importing cisplatin, VRACs directly respond to osmotic stress by trafficking osmolytes across the plasma membrane to regulate cell volume (*38*). Given that NPEPPS is found in complex with LRRC8A, we hypothesized that NPEPPS is a negative regulator of VRAC transport function. Thus, we tested the impact of NPEPPS manipulations on osmolytes known to be transported through VRACs. Using untargeted metabolomics, we found that short hairpin RNA (shRNA)–mediated suppression of NPEPPS significantly decreased the levels of intracellular taurine, hypotaurine, creatine, phosphocreatine, and several other amino acids (Fig. 2A and table S2), which are known to be exported via VRACs (*22*, *38*, *39*). In addition, intracellular taurine levels were reduced even further when cells with knockdown of NPEPPS were also treated with 10 μM cisplatin (Fig. 2A). Absolute quantification of taurine in T24 cells with NPEPPS knockdown at 24 hours confirmed these findings (Fig. 2A). These results support the NPEPPS-mediated regulation of the VRACs and suggest that cisplatin further stimulates channel activity when NPEPPS expression is decreased, allowing for increased taurine export.

To evaluate the impact of each VRAC subunit (LRRC8A-E) on cisplatin import, we measured intracellular platinum (Pt-195) accumulation in tens of thousands of KU1919 cells by performing cytometry by time-of-flight (CyTOF) following cisplatin treatment (10 μM for 4 hours) with and without small interfering RNA (siRNA)–mediated LRRC8A-E subunit depletion. LRRC8A-E protein knockdown was validated by Western blot in cell lysates collected within 24 hours of CyTOF analysis (fig. S6). Intracellular cisplatin was decreased with depletion of LRRC8A (*P*.adj < 0.0001) and LRRC8D (*P*.adj = 0.0007) but not with loss of LRRC8B, LRRC8C, or LRRC8E (Fig. 2B). Given that LRRC8A had the strongest effect on platinum uptake and that it is the “obligate subunit” of the VRAC for channel function (*32*), all subsequent experiments testing VRAC function were performed with LRRC8A manipulations.

To determine the functional relationship between NPEPPS and VRACs on intracellular cisplatin import, we performed a series of siRNA experiments targeting *NPEPPS* and/or *LRRC8A* (Fig. 2C). We found that the knockdown of NPEPPS consistently increased the import of cisplatin. As expected, knockdown of LRRC8A resulted in decreased intracellular cisplatin (KU1919 *P*.adj < 0.0001; T24 *P*.adj = 0.03; 5637 *P*.adj < 0.0001), but knockdown of NPEPPS in combination with LRRC8A knockdown showed no effect across all three BCa cell lines (Fig. 2C). We performed the same siRNA experiments on the gemcitabine and cisplatin (GemCis)–resistant derivative cells. As expected, depletion of LRRC8A did not result in additional resistance as these cells are already resistant, whereas NPEPPS knockdown alone resulted in increased intracellular cisplatin (fig. S7). As we found in the nonresistant parental cells, NPEPPS knockdown had no effect when LRRC8A was also depleted (fig. S7). We then repeated these experiments with carboplatin (100 μM for 4 hours), in parental and GemCis-resistant KU1919 and T24 cell lines. Consistent with our observations of cisplatin, we saw that intracellular carboplatin accumulation increased in parental and resistant cells with NPEPPS knockdown, and this effect was lost with the knockdown of LRRC8A (fig. S8).

We previously showed that increased intracellular cisplatin via NPEPPS knockdown in treatment-resistant BCa cells resulted in sensitization to cisplatin (*12*). Here, we evaluated the impact of intracellular cisplatin concentrations on LRRC8A protein expression and DNA damage. In platinum-resistant KU1919, T24, and 5637 cells, after 48 hours of 10 μM cisplatin treatment, we found unchanged levels of LRRC8A in shRNA-mediated NPEPPS depletion and shRNA controls (Fig. 2D). NPEPPS-depleted cells treated with cisplatin showed elevated DNA damage, as indicated by increased phosphorylation of histone H2AX (phospho-γ-H2AX), compared to controls (Fig. 2D). These results point to NPEPPS regulating intracellular cisplatin concentrations via VRACs.

### NPEPPS colocalizes with LRRC8A at the cell membrane

**Fig. 3.**
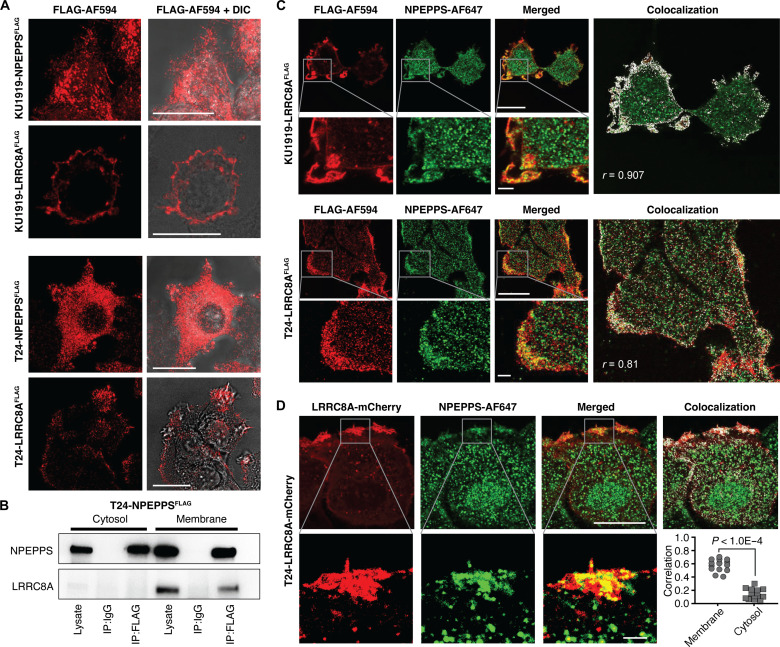
Subcellular localization of NPEPPS protein and VRAC. (**A**) Confocal microscopy images illustrating the cellular localization of NPEPPS and LRRC8A via NPEPPS^FLAG^ or LRRC8A^FLAG^ in both KU1919 and T24 cells. Shown with and without differential interference contrast (DIC). Scale bars, 20 μm. (**B**) Immunoblots for NPEPPS and LRRC8A after FLAG affinity pull-down from NPEPPS^FLAG^ in cytosolic and membranal fractions of T24 cell lysates. (**C**) Dual staining of LRRC8A and NPEPPS in LRRC8A^FLAG^ KU1919 and T24 cells using anti-FLAG primary and Alexa Fluor (AF) 594 secondary antibodies for LRRC8A and anti-NPEPPS primary and AF647 secondary antibodies for NPEPPS. Scale bars, 20 μm [for nonzoomed images (top row)] and 2 μm [for zoomed images (bottom row)]. (**D**) Confocal microscopy showing colocalization of NPEPPS and LRRC8A in LRRC8A-mCherry reporter-containing T24 cells stained with anti-NPEPPS primary and AF647 secondary antibodies. Scale bars, 20 μm [for nonzoomed images (top row)] and 2 μm [for zoomed images (bottom row)]. NPEPPS-LRRC8A colocalization was calculated in ImageJ by PCC (r or Correlation), and statistical significance was evaluated by unpaired *t* test.

We engineered BCa cell lines KU1919 and T24 to express NPEPPS^FLAG^ or LRRC8A^FLAG^ and analyzed the subcellular distribution of each protein. Using fluorescently labeled anti-FLAG antibodies and confocal microscopy, we found that NPEPPS protein was ubiquitous throughout the cell. In contrast, LRRC8A was primarily localized to the plasma membrane (Fig. 3A), which is consistent with a previous report (*38*). To support our microscopy findings, we separated the cytosolic and membrane cell fractions of T24-NPEPPS^FLAG^ cells by affinity chromatography and performed anti-FLAG IP and Western blotting for NPEPPS and LRRC8A. NPEPPS was found in both compartments, whereas LRRC8A and the NPEPPS-LRRC8A interaction complex were found only in the membrane fraction (Fig. 3B).

Given our observed colocalization of NPEPPS and LRRC8A at the plasma membrane, we hypothesized that NPEPPS interacts with the VRAC’s intracellular (LRR) domains to mediate cisplatin import. We used immunofluorescence to stain cells expressing LRRC8A^FLAG^ and endogenous NPEPPS^WT^ using anti-FLAG and anti-NPEPPS antibodies in combination. The results in both KU1919 and T24 cells demonstrate significant colocalization, defined as Pearson’s correlation coefficient (PCC) > 0.5, of NPEPPS with LRRC8A^FLAG^ at the cell membrane (Fig. 3C). We also expressed fluorescence-based reporter-tagged LRRC8A (LRRC8A^mCherry^) in T24 cells and stained NPEPPS with an endogenous anti-NPEPPS antibody. In these cells, LRRC8A was again found to be primarily located at the plasma membrane, with minimal cytoplasmic localization (Fig. 3D). Consistent with our previous observations, we detected higher colocalization of NPEPPS and LRRC8A at the cell membrane than in the cytosolic compartment (*P* < 0.0001 by unpaired *t* test) (Fig. 3D).

### Pharmacological inhibition of NPEPPS disrupts the NPEPPS-LRRC8A interaction

**Fig. 4.**
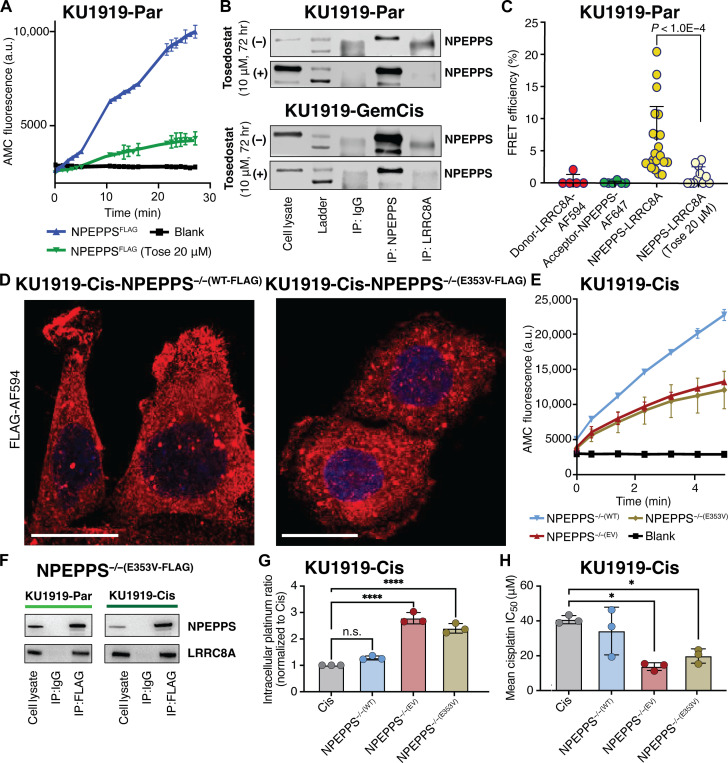
NPEPPS enzymatic function is critical for the NPEPPS-LRRC8A interaction. (**A**) Quantification of NPEPPS enzymatic activity on the H-Leu-AMC substrate with vehicle (PBS, 0 μM) or tosedostat (20 μM) treatment in NPEPPS^FLAG^ KU1919 parental cells. a.u., arbitrary units. (**B**) Immunoblot of NPEPPS and LRRC8A in KU1919 parental and GemCis-resistant cells after IP with native NPEPPS or LRRCA antibodies following 72 hours of PBS control (0 μM) or tosedostat (10 μM) treatment. (**C**) Quantification of NPEPPS-LRRC8A colocalization by FRET-AB in LRRC8A^FLAG^ KU1919 parental cells with vehicle (PBS, 0 μM) or tosedostat (20 μM) treatment. The relative quantification is reported as FRET efficiency (%) and statistical comparisons were made using the Mann-Whitney *U* test. (**D**) Immunofluorescence confocal microscopy of NPEPPS^FLAG^ constructs [NPEPPS^−/−(WT-FLAG)^ and NPEPPS^−/−(E353V-FLAG)^] in cisplatin-resistant KU1919 cells. Scale bars, 20 μm. (**E**) Quantification of NPEPPS enzymatic activity on the H-Leu-AMC substrate in KU1919-Cis cells expressing NPEPPS^−/−(WT-FLAG)^ or NPEPPS^−/−(E353V-FLAG)^. (**F**) Immunoblots of NPEPPS and LRRC8A following anti-FLAG IP in parental and cisplatin-resistant KU1919 cells expressing NPEPPS^−/−(WT-FLAG)^ or NPEPPS^−/−(E353V-FLAG)^. (**G**) Intracellular cisplatin concentrations were measured by CyTOF in triplicate experiments in WT KU1919-Cis cells or KU1919-Cis cells expressing NPEPPS^−/−(WT-FLAG)^ or NPEPPS^−/−(E353V-FLAG)^. Cisplatin concentrations were normalized to the unmodified cisplatin-resistant KU1919 cells, and comparisons were made using one-way ANOVA (****P < 0.0001). (**H**) Cisplatin IC_50_ was measured in technical and biological triplicate by IncuCyte Zoom analysis over 120 hours of treatment in WT KU1919-Cis cells or KU1919-Cis cells expressing NPEPPS^−/−(WT-FLAG)^ or NPEPPS^−/−(E353V-FLAG)^. Results are reported as the mean IC_50_ ± SD. Comparisons were made using one-way ANOVA (*P < 0.05).

We have shown that the nonspecific aminopeptidase inhibitor tosedostat can reverse NPEPPS-mediated cisplatin resistance in BCa cells (*12*). Integrating this drug into our mechanistic studies, we first assessed the ability of tosedostat to inhibit the catalytic activity of NPEPPS using a reporter assay that generates a fluorescent signal on the cleavage of substrate Leu-AMC (l-leucine-7-amido-4-methylcoumarin, a typical substrate of NPEPPS and other leucyl aminopeptidases). We found that peptide cleavage in KU1919 cells with NPEPPS^FLAG^ was inhibited by 20 μM tosedostat (Fig. 4A and fig. S9A). We then assessed the impact of tosedostat treatment on the NPEPPS-LRRC8A interaction by performing IP-immunoblot analysis on protein lysates from BCa cells before and after tosedostat treatment. We found that treating with 10 μM tosedostat for 72 hours reduced NPEPPS-LRRC8A coprecipitation in both NPEPPS^FLAG^ anti-FLAG IP and NPEPPS^WT^ anti-NPEPPS IP with endogenous antibody, without altering the total levels of NPEPPS protein (Fig. 4B and fig. S9, B and C).

To further characterize the impact of tosedostat on the NPEPPS-LRRC8A interaction, we used Förster resonance energy transfer (FRET)–acceptor bleaching (AB)-based quantification to evaluate the NPEPPS-LRRC8A interaction on the cell surface before and after tosedostat treatment. In the untreated group, FRET occurred between NPEPPS and LRRC8A, indicating a direct interaction in the functional complex. We found that FRET efficiency decreased with 20 μM tosedostat treatment, again suggesting reduced formation of the NPEPPS-LRRC8A complex (Fig. 4C and fig. S10). Last, we assessed the platinum uptake phenotype in BCa cells and used CyTOF to show that adding 20 μM tosedostat treatment significantly increased the intracellular platinum accumulation in KU1919-GemCis cells compared to those treated with cisplatin alone (fig. S11). Furthermore, depleting LRRC8A with siRNA effectively ablated this increase compared to control siRNA (fig. S11).

### NPEPPS requires enzymatic activity to bind to LRRC8A and regulate cisplatin import

With results showing that the NPEPPS-LRRC8A interaction can be pharmacologically targeted to improve chemosensitivity, we asked whether this functional interaction depends on the enzymatic and substrate binding properties of NPEPPS. We leveraged a previously validated mutant of NPEPPS to explore this question. The E353V (AA>TG) mutation in NPEPPS causes loss of catalytic activity without disrupting substrate binding (*40*, *41*). We used this mutation in the context of CRISPR-edited NPEPPS^−/−^ cell lines to test the contributions of NPEPPS’s catalytic activity and substrate binding to its interactions with VRAC subunit protein LRRC8A.

To ensure that the mutant construct was adequately expressed and localized as expected, we used a fluorescent anti-FLAG antibody and found that the protein constructs [NPEPPS^−/−(WT-FLAG)^ and NPEPPS^−/−(E353V-FLAG)^] were distributed across cells (Fig. 4D). We then tested the enzymatic function of each construct using the Leu-AMC reporter assay. Purified protein lysates from NPEPPS^−/−(WT-FLAG)^ cells showed the expected enzymatic activity, whereas the NPEPPS^−/−(EV)^ and NPEPPS^−/−(E353V-FLAG)^ lysates had consistently low enzymatic function as expected (Fig. 4E). To dissect the distinct catalytic and binding roles of NPEPPS in the interaction with LRRC8A, we assessed the NPEPPS^−/−(E353V-FLAG)^ construct by IP-FLAG-immunoblot analysis. Intriguingly, our results in KU1919-Cis (Fig. 4F) and T24-Cis (fig. S12) cells revealed that the catalytic-dead NPEPPS^−/−(E353V-FLAG)^ construct can still interact with LRRC8A (Fig. 4F).

After validating the mutant construct localization, enzymatic activity, and interactions with LRRC8A, we explored the functional impact of the point mutation on cisplatin uptake and growth inhibition in BCa cells. We previously reported that shRNA-mediated depletion of NPEPPS in resistant KU1919 and T24 cells resulted in increased intracellular cisplatin accumulation (*12*). Using an antibiotic (puromycin)–free CRISPR system to avoid inducing NPEPPS, we confirmed that NPEPPS knockout (KO) in cisplatin-resistant cells (KU1919-Cis-NPEPPS^−/−^) resulted in the predicted increase in intracellular cisplatin, and subsequent addback of FLAG-tagged NPEPPS [KU1919-Cis-NPEPPS^−/−(WT-FLAG)^] rescued intracellular cisplatin levels back to those seen in the wild-type (WT) cisplatin-resistant cells (Fig. 4G). In contrast, NPEPPS^−/−(E353V-FLAG)^ or NPEPPS^−/−(EV)^ did not affect intracellular platinum levels compared to NPEPPS^−/−^ (Fig. 4G).

We assayed BCa cell growth inhibition in a cisplatin dose course for 120 hours (table S3). Differences in mean median inhibitory concentrations (IC_50_’s) were evaluated with one-way analysis of variance (ANOVA) test using unmodified KU1919-Cis-NPEPPS^WT^ cells as a control group. As expected, NPEPPS^−/−(EV)^ cells were significantly sensitized to cisplatin (IC_50_: 13.8 μM) compared to unmodified NPEPPS^WT^ control cells (40.7 μM, *P*.adj = 0.02) whereas NPEPPS^−/−(WT-FLAG)^ cells (34.2 μM) showed resistance levels near those of the unmodified controls (Fig. 4H). Consistent with the exposure levels seen in CyTOF, the NPEPPS^−/−(E353V-FLAG)^ (19.8 μM) mutant construct showed similar resistance phenotypes to NPEPPS^−/−(EV)^ control cells (Fig. 4H). These results highlight the critical role of NPEPPS’s catalytic activity in reducing cisplatin import through the VRACs.

### Loss of NPEPPS catalytic activity sensitizes BCa tumors to cisplatin in vivo

**Fig. 5.**
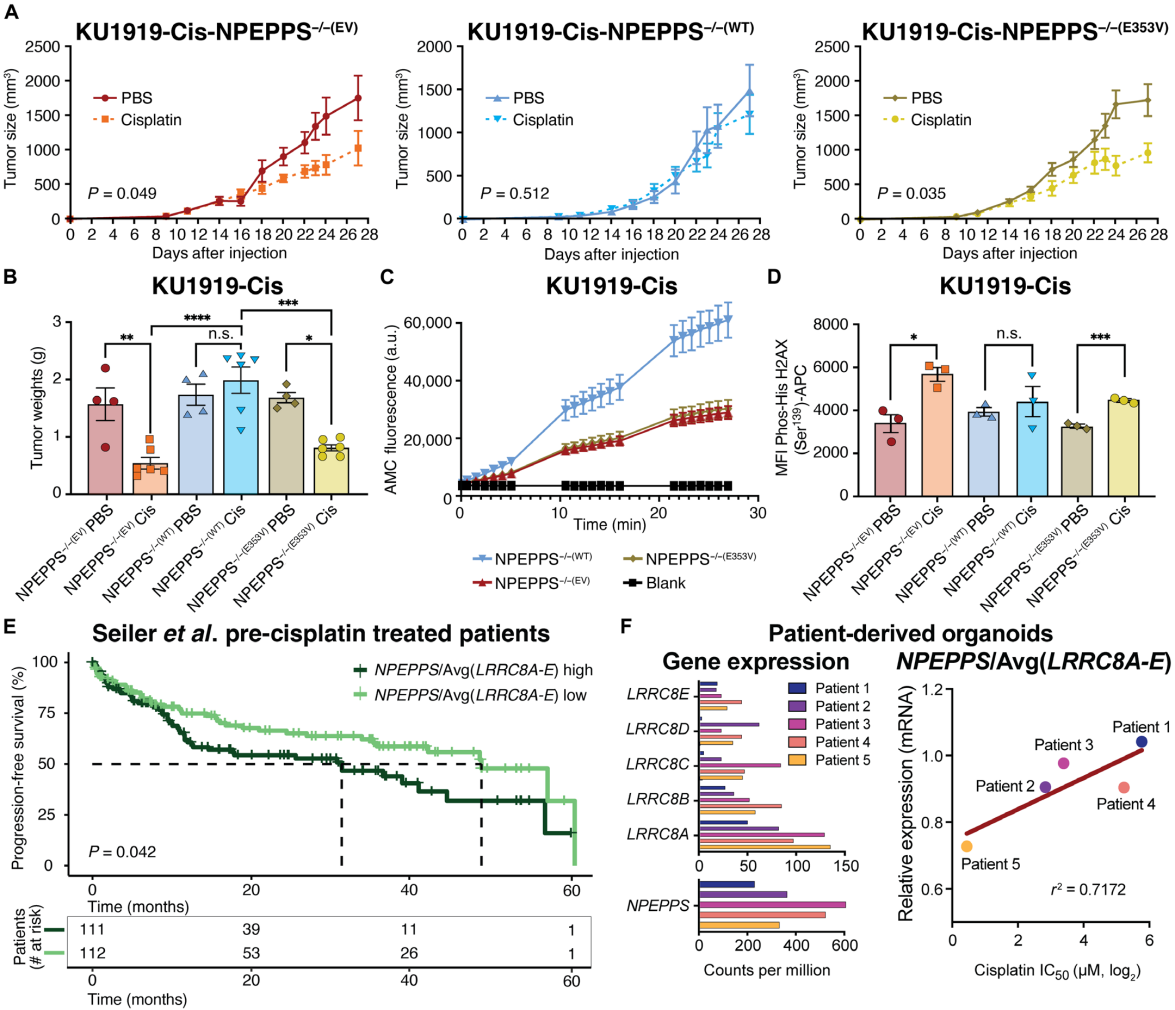
NPEPPS enzymatic function regulates cisplatin response in vivo. (**A**) Mice were injected with 4 × 10^6^ cells in each flank to generate tumors using KU1919-Cis cells with NPEPPS^−/−(WT-FLAG)^ or NPEPPS^−/−(E353V-FLAG)^ expression. When engrafted tumors reached 100 mm^3^, mice were randomized to control or treatment groups and received either cisplatin (2 mg/kg; by intraperitoneal injection three times per week) or PBS control (equal volume of saline by intraperitoneal injection three times per week). Tumor size was measured with calipers every 2 to 3 days throughout the duration of treatment. Differences in growth rates by tumor genotype were evaluated by a two-way ANOVA mixed effects model. (**B**) Wet tumor weights were measured for each group. Comparisons were made using a one-way ANOVA (*P < 0.05; **P < 0.01; ***P < 0.001; ****P < 0.0001). (**C**) Fluorescence-based enzyme assay to quantify the catalytic activity of NPEPPS across the cells isolated from mouse tumors using H-Leu-AMC as the substrate. (**D**) DNA damage was measured by flow cytometry–based quantification of phosphorylated histone H2AX. Comparisons were made using a one-way ANOVA (*P < 0.05; ***P < 0.001). (**E**) Stratification of patient survival by above-median or below-median expression of the ratio of *NPEPPS*/Avg(*LRRC8A-E*) in BCa tumor samples before cisplatin treatment. Analysis of survival differences was conducted in R, and statistical significance was evaluated by Cox proportional hazard ratios and the log-rank test. Time to median survival is indicated by a dashed line. (**F**) Patient-derived organoids were evaluated for mRNA expression and cisplatin IC_50_. Left: Average counts per million mRNA expression of VRAC subunits *LRRC8A-E* and *NPEPPS* in each patient-derived organoid. Right: The correlation of *NPEPPS*/Avg(*LRRC8A-E*) expression with cisplatin sensitivity is plotted (*r*^2^ = 0.7172). Organoids from patient 1 were derived from a cystectomy sample, and the patient was not given neoadjuvant chemotherapy (NAC). Organoids from patients 2 to 5 were derived from the transurethral resection of bladder tumor samples before the patients received NAC.

We validated the in vitro phenotypic effects described above by developing mouse xenograft models with our NPEPPS^−/−(E353V-FLAG)^ and NPEPPS^−/−(WT-FLAG)^ constructs, as well as control NPEPPS^−/−(EV)^ cells, to assess the cisplatin response of each tumor in vivo. Tumor volumes in both the cisplatin-treated NPEPPS^−/−(EV)^ and NPEPPS^−/−(E353V-FLAG)^ mutant groups were smaller than those in the saline group (*P* < 0.05 by two-way ANOVA mixed effects model) (Fig. 5A). In contrast, the NPEPPS^−/−(WT-FLAG)^ tumors were larger, consistent with our in vitro findings. Tumor weights at the study endpoint corroborated these observations, mirroring patterns seen in tumor growth kinetics (Fig. 5B). Tumor cells were sorted by flow cytometry with anti–human leukocyte antigen (HLA) and anti-FLAG antibodies, and NPEPPS-addback constructs were validated by next-generation sequencing. The Leu-AMC cleavage assay described above found that tumor cells isolated from NPEPPS^−/−(EV)^ and NPEPPS^−/−(E353V-FLAG)^ showed lower catalytic activity compared to those from NPEPPS^−/−(WT-FLAG)^ tumors, supporting our prior findings that NPEPPS catalytic activity correlates with treatment response (Fig. 5C). We then analyzed tumors from each group by flow cytometry, measuring phospho-γ-H2AX activation as a marker for DNA damage (*42*), a key measure of tumor cisplatin exposure. The NPEPPS^−/−(E353V-FLAG)^ cells again behaved similarly to the NPEPPS^−/−(EV)^, displaying significantly more DNA damage and increased susceptibility to cisplatin-induced cell death (Fig. 5D). In contrast, cisplatin-treated NPEPPS^−/−(WT-FLAG)^ tumor cells exhibited lower levels of phospho-γ-H2AX, indicative of reduced exposure and sensitivity to cisplatin (Fig. 5D). These in vivo findings strengthen our in vitro data, suggesting that the NPEPPS-LRRC8A interaction is a critical targetable node in cisplatin resistance.

### *NPEPPS-VRAC* gene expression stratifies BCa cisplatin response in patients and patient-derived organoids

We tested the clinical relevance of the *NPEPPS-VRAC* interaction by analyzing BCa patient data and patient-derived organoid models. First, to evaluate the relationship between gene expression (GE) and progression-free survival in patients with cisplatin-treated BCa, we used GE data from BCa tumor samples collected before cisplatin treatment (*43*). Given the large sequence homology between the VRAC subunits (*44*), we evaluated *NPEPPS* and *LRRC8A-E* individually and in combination, including calculating averages of *LRRC8A* and *LRRC8D*, *LRRC8A-E*, and ratios of *NPEPPS* to individual or averaged subunit expression. *NPEPPS* and the *VRAC* subunit genes showed variable predictive ability for progression-free survival using median stratification to define Cox proportional hazard ratios. We found that the most robust and consistent signal was median stratification using the ratio of *NPEPPS* to the average expression of all *VRAC* subunits [Avg(*LRRC8A-E*)]. Patients in the Seiler *et al.* dataset with higher *NPEPPS*/Avg(*LRRC8A-E*) expression ratios had worse progression-free survival than those with lower ratios (*P* = 0.042) (Fig. 5E). We further evaluated the potential correlations between GE and clinical features such as age, sex, chemotherapy type, and disease stage in this dataset and found no significant differences in the *NPEPPS*/Avg(*LRRC8A-E*) ratio (*t* test for age and sex; one-way ANOVA for chemotherapy type and disease stage). To support this analysis, we investigated a dataset of GE and tumor response reported in Taber *et al.* (*45*) and found a statistically significant relationship between *NPEPPS*/Avg(*LRRC8A-E*) expression ratios and Response Evaluation Criteria in Solid Tumors scoring categories of complete response compared to stable disease (*P* = 0.0148). The ratio was lowest in the complete response category (mean ratio = 3.7) and elevated in the stable disease group (mean ratio = 4.5) (fig. S13). Last, using The Cancer Genome Atlas (TCGA) Project BCa cohort (*46*), we evaluated *NPEPPS*/Avg(*LRRC8A-E*) in BCa tumors where the patient did not have a recorded platinum-based treatment (*n* = 203) and found no difference in survival outcomes (*P* = 0.57), suggesting that *NPEPPS*/Avg(*LRRC8A-E*) is not a prognostic marker but is predictive of platinum-based chemotherapy response.

A similar pattern was found in patient-derived BCa organoids (Fig. 5F) (*12*). Treatment response was evaluated by growth inhibition analysis to establish IC_50_ values of cisplatin. Using RNA sequencing (RNA-seq) data, we found that higher cisplatin IC_50_ values correlated with higher levels of *NPEPPS*/Avg(*LRRC8A-E*) ratios (*r*^2^ = 0.717 by linear regression) (Fig. 5F). Consistent with the patient cohort (Fig. 5E), all organoids were derived from patient tumors before platinum-based chemotherapy was administered. These results support our in vitro, in vivo, and patient outcome data and the translational potential of our work.

### NPEPPS-mediated platinum resistance is relevant in non-BCa cell types and patient cohorts

**Fig. 6.**
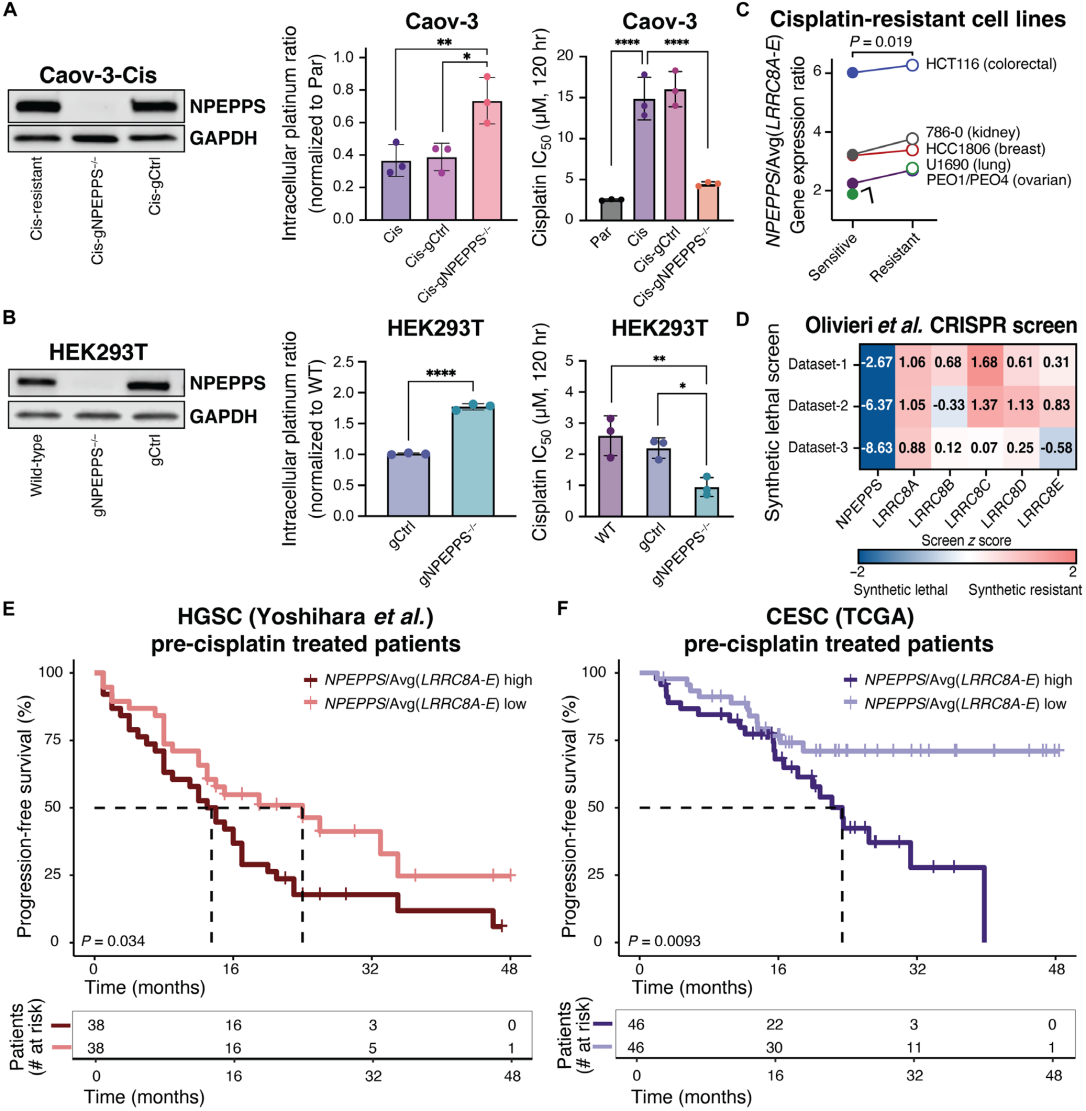
NPEPPS regulates platinum chemotherapy import and efficacy beyond BCa. (**A**) Immunoblots of NPEPPS in cisplatin-resistant Caov-3 cells (Caov-3-Cis) with WT NPEPPS (Caov-3-Cis-gCtrl) or KO of NPEPPS (Caov-3-Cis-gNPEPPS^−/−^). Intracellular cisplatin concentrations were measured by CyTOF in triplicate experiments in WT, chemotherapy-sensitive Caov-3-Par cells, WT, chemotherapy-resistant Caov-3-Cis cells, or engineered Caov-3-Cis cells with control (-gCtrl) or NPEPPS KO (-*NPEPPS*^−/−^). Cisplatin concentrations were normalized to unmodified cisplatin-sensitive Caov-3-Par cells, and comparisons were made using one-way ANOVA (*P < 0.05; **P < 0.01). Cisplatin IC_50_ was measured in technical and biological triplicate in WT Caov-3-Par, Cis, Cis-gCtrl, or Cis-gNPEPPS^−/−^ cells. Results are reported as the mean IC_50_ ± SD. Comparisons were made using one-way ANOVA (****P < 0.0001). (**B**) Immunoblots of NPEPPS in WT HEK293T cells, HEK293T-gCtrl cells, or HEK293T-gNPEPPS^−/−^ cells. Intracellular cisplatin concentrations were measured by CyTOF, and comparisons were made using one-way ANOVA (****P < 0.0001). Cisplatin IC_50_ was measured in technical and biological triplicate and reported as the mean IC_50_ ± SD. Comparisons were made using one-way ANOVA (*P < 0.05; **P < 0.01). (**C**) GE data from paired cisplatin-sensitive and cisplatin-resistant cancer cell lines representing colorectal, kidney, breast, lung (*48*), and ovarian (*49*) cancers were evaluated for the expression ratio of *NPEPPS*/Avg(*LRRC8A-E*). The comparison was made using a paired *t* test. (**D**) Synthetic lethal and synthetic resistant z scores of NPEPPS and LRRC8A-E across three cisplatin datasets were evaluated by CRISPR screening in the RPE1 cell line and retrieved from the Olivieri *et al.* CRISPR screen repository (*50*). (**E**) Stratification of patient survival by above-median or below-median expression of the ratio of *NPEPPS*/Avg(*LRRC8A-E*) in tumor samples from HGSC [Yoshihara *et al.* (*51*)] and CESC [TCGA (*46*)] prior to platinum-based treatment. Time to median survival is indicated by a dashed line (omitted where survival is >50% at all time points), and comparisons were made using the log-rank test.

Cisplatin-based chemotherapy remains the standard of care for numerous cancers (*1*, *3*, *4*). To demonstrate the broader applicability of our work beyond BCa, we tested the impact of knocking out NPEPPS in cisplatin-resistant high-grade serous carcinoma (HGSC) cell line Caov-3 (*47*). We generated Caov-3-Cis-NPEPPS^−/−^ cells (fig. S14) and then used CyTOF and dose-response assays to evaluate intracellular platinum accumulation and growth inhibition. As we observed in BCa, NPEPPS KO in cisplatin-resistant HGSC cells led to a significant increase in intracellular cisplatin and a significant decrease in the cisplatin IC_50_ (Fig. 6A). We repeated this process in human embryonic kidney (HEK) 293T cells, the cell type reported in the BioPlex database to have NPEPPS-LRRC8A-E interactions (*35*), to establish an NPEPPS-KO phenotype in noncancer cells. We found that, even in this context, cells with NPEPPS loss had higher platinum accumulation by CyTOF and increased sensitivity to cisplatin compared to WT HEK293T cells (Fig. 6B). We compiled previously published reports of GE in cancer cell lines before and after cisplatin exposure and found multiple cases in which the expression ratio of *NPEPPS*/Avg(*LRRC8A-E*) was significantly elevated in cisplatin-resistant cells, including in cancers from the colorectum, kidney, breast, and lung [retrieved from Carroll *et al.* (*48*)] and ovarian tissues [retrieved from Gallon *et al.* (*49*)] (Fig. 6C).

Olivieri *et al.* evaluated synthetic lethal and synthetic resistant interactions in RPE1 (noncancerous) cells by genome-wide CRISPR screening across 27 genotoxic drugs, including cisplatin, concatenating triplicate independent experiments (*50*). Consistent with our prior findings, these data show strong synthetic lethal interaction scores with NPEPPS and cisplatin and synthetic resistant interactions with each of the five VRAC subunit proteins LRRC8A-E (Fig. 6D). We also searched publicly available datasets to evaluate the significance of the NPEPPS-VRAC interaction in patient survival beyond BCa. We found that patients who would go on to receive cisplatin therapy (pretreatment tumor samples) with the mRNA expression ratio *NPEPPS*/Avg(*LRRC8A-E*) above the median had significantly worse progression-free survival outcomes compared to those with expression ratios below the median, both in a cohort of patients with HGSC from Yoshihara *et al.* (*51*) (*P* = 0.034) and in the TCGA Pan-Cancer Atlas 2018 cohort of patients with cervical squamous cell carcinoma (CESC) (*46*) (*P* = 0.0093) (Fig. 6E). We also found a statistically significant elevation in *NPEPPS*/Avg(*LRRC8A-E*) GE ratio in clinical tumor stages T2 and T3/T4 (advanced disease) compared to Tis/T1 (early-stage disease) (*P* = 0.003 and *P* = 0.0149 by one-way ANOVA) as well as a higher incidence of metastasis in patients with above-median GE ratio (*P* = 0.0032 by *t* test) (fig. S15). As in the TCGA BCa cohort, we found that patients in the TCGA CESC dataset who did not have a record of platinum-based treatment (*n* = 147) had no significant differences in progression-free survival based on their *NPEPPS*/Avg(*LRRC8A-E*) expression levels (*P* = 0.26). Together, these results underscore that the mechanism of the NPEPPS regulation of VRACs and their platinum import activity is consistent across multiple cancer contexts.

## DISCUSSION

We uncover a native, intracellular regulator of platinum import by VRACs and discover its mode of action. Using BCa as a model, we found that the VRAC subunit LRRC8A is a critical NPEPPS interactor regulating VRAC-mediated cisplatin import. We have shown the importance of this interaction in multiple contexts, including various types of cancer and noncancer cell lines, in the experimental setting, and in patient outcomes with platinum-based therapy. Whereas NPEPPS was recognized as a ubiquitous M1 class aminopeptidase participating in various cellular processes and controversially linked to the regulation of tauopathies (*52*–*58*), this enzyme now emerges as a pivotal, druggable interaction partner governing the response to cisplatin by regulating VRAC activity. Furthermore, analysis of the mutant NPEPPS variant underscores the crucial role of its enzymatic domain in mediating this interaction. Our intriguing finding that catalytic-dead NPEPPS can complex with LRRC8A but cannot drive resistance through the interaction provides a mechanistic foundation for exploring the role of the aminopeptidase activity of NPEPPS on VRACs.

NPEPPS mutations are remarkably infrequent across ~70,000 human tumors (0.4%) (*59*–*61*), yet NPEPPS-KO mouse models are viable. Together, these data support NPEPPS as a stable, druggable target in treatment-resistant cancer (*41*, *62*). This genetic stability, coupled with the essential nature of the VRAC channels in normal biology, may offer unique and important advantages by limiting the possible emergence of resistance to specific allosteric or competitive inhibitors of this NPEPPS-VRAC interaction. Such inhibitors could be applied alongside cisplatin combination regimens such as GemCis, MVAC (methotrexate-vinblastine-adriamycin-cisplatin), or GemCis-immunotherapy treatment to boost platinum import in tumors and improve patient survival. Furthermore, patients deemed ineligible for traditional GemCis therapy or immunotherapy could be candidates for dose-decreased GemCis–anti-NPEPPS therapy as the addition of an NPEPPS inhibitor may lower the effective dose required to achieve similar intracellular platinum levels in cancer cells while offering some tumor specificity given that NPEPPS levels are higher in cancer cells compared to normal cells (*12*).

Our work has limitations and leaves several research areas for future investigation. First, findings in vitro and in vivo and in patient-derived organoids only partially represent the complexities of patient biology. In addition, the mechanism of action of tosedostat remains to be understood entirely. Prior studies demonstrate a promising safety profile yet heterogeneous, context-specific patient responses (*63*–*68*). It is important to note that tosedostat also targets other aminopeptidases, including aminopeptidase N and leucyl aminopeptidases, and this may account for some of the observed effects when used in the absence of platinum agents (*51*, *52*, *54*–*56*). The data presented here does not fully illuminate the mechanism by which NPEPPS alters LRRC8A and, in turn, reduces VRAC activity. Cocrystallization, scanning mutagenesis, including truncation of the LRRC8A intracellular LRR domain, and other techniques will be the subject of future studies to define the precise biophysical interaction between NPEPPS and LRRC8A. Understanding what other aspects of VRAC function, in addition to platinum import, are regulated by NPEPPS catalytic activity, such as channel formation, substrate specificity, or protein degradation, will also be insightful.

In conclusion, this study unveils a previously unknown complexation event between NPEPPS and LRRC8A proteins, collectively establishing a compelling rationale for targeting NPEPPS to combat cisplatin resistance and enhance chemotherapy efficacy in cancer. This work advances our understanding of drug transport mechanisms and sheds light on the intricate pathways underlying platinum drug resistance development. Our findings open an untapped vista for therapeutic development into targetable platinum resistance mechanisms.

## MATERIALS AND METHODS

### Cell culture

All human BCa cell lines and HGSC cell line Caov-3 were obtained from the Resistant Cancer Cell Line (RCCL) Collection and were grown in Iscove’s modified Dulbecco’s medium (IMDM) with 10% fetal bovine serum (FBS). Cells were passaged every 2 to 3 days. Resistance to cisplatin was confirmed at the reported resistance dose from the RCCL and as described in (*12*). HEK293T cells were cultured in Dulbecco’s modified Eagle’s medium (DMEM) (high glucose) supplemented with 0.1 mM nonessential amino acids (NEAA), 6 mM l-glutamine, and 1 mM sodium pyruvate with 10% FBS added. Lentivirus production used 293FT cells (Thermo Fisher Scientific), which were maintained in DMEM (high glucose) supplemented with 0.1 mM NEAA, 6 mM l-glutamine, 1 mM sodium pyruvate, and geneticin (500 μg/ml; G418) with 10% FBS added. Cells were routinely monitored for mycoplasma and confirmed negative multiple times during this study using MycoAlert (Lonza). All cells were grown at 37°C with 5% CO_2_ in a humidified incubator.

All molecular characterization efforts (enzymatic activity assays, IP, microscopy, and MS proteomics) were performed on cells from independent passages and in drug-free, complete media to identify stable molecular changes rather than treatment-induced transient response. Cells were routinely passaged through drug-containing media at the resistant doses (table S3) to confirm that resistance was maintained, and early passage cells were used whenever possible.

### Proteomics

#### Sample preparation

All cell lines were grown for several passages in IMDM + 10% FBS without antibiotics, tosedostat, or cisplatin and then seeded at 100,000 to 200,000 cells per well and grown for 48 hours. Approximately 48 hours after seeding cells, the supernatant was aspirated, and cells were washed three times with cold phosphate-buffered saline (PBS). Cells were lysed in 100 μl of 8 M urea and 50 mM tris-HCl (pH 8.0). Lysates were transferred to prechilled 1.5-ml microcentrifuge tubes and centrifuged at 15,000 relative centrifugal force (RCF) for 10 min to pellet. The supernatant was then transferred to a clean, prechilled tube and frozen. Lysate replicates were collected in independent triplicate from different passages. Cell pellets were lysed in 8 M urea supplemented with 0.1% RapiGest MS-compatible detergent. DNA was sheared using probe sonication, and protein concentration was estimated by bicinchoninic acid (BCA) protein assay (Pierce, Thermo Fisher Scientific). A total of 30 μg of protein per sample was aliquoted, and samples were diluted to a <2 M urea concentration using 200 mM ammonium bicarbonate while also undergoing reduction with dithiothreitol (DTT) (10 mM) and then alkylation with Iodoacetamide (100 mM). The pH of diluted protein lysates was verified as between 7 and 8, and samples were digested with a sequencing-grade Trypsin/Lys-C enzyme (Promega) in the presence of 10% acetonitrile for 16 hours at 37°C. Samples were acidified, adding formic acid to 1%, and speed vac dehydration was used to evaporate acetonitrile. Peptides were desalted on C18 tips (Nest Group) and dried to completion. Before MS, peptides were resuspended in a 0.1% formic acid solution at a concentration of 0.5 μg/μl with 1:40 synthetic iRT reference peptides (Biognosys).

#### Data acquisition and analysis

MS was performed on an Orbitrap Fusion Lumos Tribrid (Thermo Fisher Scientific) mass spectrometer interfaced with a microflow-nanospray electrospray ionization source (Newomics, IS-T01) coupled to an UltiMate 3000 ultra-high-pressure chromatography system with 0.1% formic acid in water as mobile phase A and 0.1% formic acid in acetonitrile as mobile phase B. Peptides were separated on a microPAC 200-cm column (Thermo Fisher Scientific) at a constant flow rate of 1.20 μl/min with 4% B for 0 to 5 min, 4 to 12% B for 5.0 to 5.2 min, 12 to 20% B for 5.2 to 55 min, 20 to 38% B for 55 to 100 min, and lastly 38 to 60% B for 100 to 120 min, all linear gradients. Source parameters were set to a voltage of 3000 V and a capillary temperature of 300°C. MS1 resolution was set to 120,000, and the automatic gain and exposure control target value for fragment spectra of 250% was used. Peptide ions were fragmented at a fixed collision energy of 30%. Fragmented ions were detected across 40 nonoverlapping data-independent acquisition (DIA) precursor windows of size 15 Da. MS2 resolution was set to 15,000 with a scan range of 200 to 2000 mass/charge ratio (*m/z*) and a maximum injection time of 25 ms. All data were acquired in profile mode using positive polarity. Peptide identification and quantification were performed using library-free search in the DIA neural networks software (*69*). Protein-level abundance was calculated using maxLFQ.

### Data normalization and visualization

Statistical analyses were performed in the language R (4.2.2). Protein abundance values were log transformed, and *P* values were calculated for each protein between the test and control groups using the function t.test assuming equal variance. FDR-adjusted *P* values were estimated using the function p.adjust from the stats package as previously described (*70*). Results were visualized using the EnhancedVolcano package in R with log_2_ FC and adjusted *P* values for *x* and *y* values.

### Metabolomics

#### Sample preparation

Cell lines were cultured for several passages in IMDM + 10% FBS (IMDM10). Before the experiment, cells were cultured in IMDM10 to ~80% confluence and then dissociated. For dissociation, cells were washed once with room temperature PBS and then incubated with PBS + 0.05% trypsin-EDTA for 10 to 15 min. Cells were neutralized with IMDM10 and then fully dissociated by gentle pipetting. After dissociation, cells were counted by Trypan blue staining and then replated at 1 × 10^6^ cells. Twenty-four hours after plating, cells were treated with either IMDM10 or IMDM10 + 10 μM cisplatin. Day 0 cell cultures were immediately processed for metabolomics analysis. To prepare cell pellets for metabolomics analysis, day 0 cells were dissociated and centrifuged at 300 RCF for 10 min at 4°C. Cells were suspended in PBS, centrifuged a second time, resuspended in PBS, and counted. Day 0 cells were centrifuged a third time, the supernatants were aspirated, and the dry cell pellets were snap frozen in liquid nitrogen and stored at −80°C until metabolite extraction. Seventy-two hours after plating, cells were processed for metabolomics analysis as described for the day 0 cell cultures.

#### Data generation and analysis

Metabolites from frozen cell pellets were extracted at 2 × 10^6^ cells/ml in ice-cold 5:3:2 MeOH:acetonitrile:water. Extractions were carried out using vigorous vortexing for 30 min at 4°C. Supernatants were clarified by centrifugation (10 min, 18,000*g*, 4°C), and 10 μl analyzed using a Thermo Fisher Scientific Vanquish UHPLC coupled to a Thermo Fisher Scientific Q Exactive mass spectrometer. Global metabolomics analyses were performed using a 5-min C18 gradient in positive and negative ion modes (separate runs) with electrospray ionization as described in (*71*, *72*). For all analyses, the MS scanned in MS1 mode across the *m/z* range of 65 to 950. Peaks were annotated with the KEGG database and integrated, and quality control was performed using Maven as described in (*73*). Data were variance stabilization normalized (*74*) and log_2_ transformed, and differential abundance calculations were done using limma (v3.44.3) (*75*) with time and treatment as covariates in the linear model.

### Cell line drug treatments

Gemcitabine (Sigma-Aldrich) and cisplatin (Sigma-Aldrich) stocks were resuspended in a 0.9% saline solution, and tosedostat (Sigma-Aldrich and BOC Sciences) was resuspended in DMSO. All stock solutions were stored protected from light and kept frozen until use. Cells were seeded in 96-well tissue culture plates for cell culture dose-response with 500 to 2000 cells per well, depending on the growth rate and experiment duration. Cells were seeded and allowed to attach overnight, followed by replacing the media with fresh, prewarmed media just before treatment. Drug dilutions were performed serially using complete media (IMDM + 10% FBS or DMEM + 10% FBS, as described above) and the associated drug treatments. Growth inhibition was measured using confluence estimates over time on the IncuCyte ZOOM (Essen Bioscience) over varying amounts of time (72 to 120 hours), depending on each experiment. Details for timing and replicates for each dose-response experiment are included in their figure legends. For co-IP assays, cells were treated for 72 hours with PBS control (0 μM) or tosedostat (10 μM) before cell lysis. For the fluorescence-based Leu-AMC assay, catalytic activity was quantified with the presence or absence of 20 μM tosedostat. For FRET-AB microscopy experiments, cells were cultured in complete media with or without 10 μM of tosedostat for 48 hours before assaying.

### Antibodies and Western blotting

Whole-cell lysates were prepared from cultured cells using a radioimmunoprecipitation assay (RIPA) lysis and extraction buffer (Thermo Fisher Scientific). Lysates from xenograft tissues were prepared using a tissue protein extraction reagent (T-PER) and glass tissue homogenizer. All lysates were prepared on ice with Halt protease, phosphatase inhibitor cocktail, and EDTA (Thermo Fisher Scientific). The protein concentration of lysates was quantified with a BCA protein assay (Pierce, Thermo Fisher Scientific). All lysates were prepared with a 4X LI-COR loading buffer with 50 μM DTT added and boiled for 10 min before gel loading. All Western blots were run using PROTEAN TGX precast 4 to 15% or 4 to 20% gradient gels (Bio-Rad) and transferred to 0.2- or 0.44-μm nitrocellulose membranes. The transfer was done for 1.5 to 2 hours in a cold Tris/Glycine buffer (Bio-Rad) with 20% methanol before blocking for 1 hour at room temperature in 5% BSA in a 1X Tris buffer (TBS-T) (Bio-Rad). Primary antibodies were diluted and incubated overnight at 4°C on a rocker. Membranes were washed three or four times in fresh TBS-T before a 1-hour room temperature incubation in an appropriate secondary antibody. Membranes were washed three to four times in TBS-T, developed with enhanced SuperSignal West Pico Plus or SuperSignal West Fempto (Thermo Fisher Scientific), and imaged using LI-COR Odyssey Fc instrument. Densitometry was performed using the LI-COR Image Studio software. Statistical comparisons using densitometry measurements were made using a one-way ANOVA with Tukey post hoc to control for the experiment-wise error rate. Antibodies used include the following: NPEPPS (Invitrogen, PA5-83788), glyceraldehyde-3-phosphate dehydrogenase (GAPDH) (Cell Signaling Technology, 5174), LRRC8A (LSBio, LS-C290818 and LS-B16989), LRRC8B (Sino Biological, 103247-T36), LRRC8C (Proteintech, 21601-1-AP), LRRC8D (Sino Biological, 104245-T32), LRRC8E (Abcam, ab201188), Phospho-Histone H2A.X (Ser^139^) (Invitrogen, MA1-2022), FLAG (Sigma-Aldrich, F1804), and anti-mouse immunoglobulin G (IgG) (Sigma-Aldrich, A9044; MP Biomedicals, 855689).

### Immunoprecipitation

IP of FLAG-tagged human BCa cell lines was carried out using Protein G Sepharose beads following the manufacturer’s protocol (GE HealthCare). Cells were lysed using a Pierce IP lysis buffer containing 25 mM tris-HCl (pH 7.4), 150 mM NaCl, 1% NP-40, 1 mM EDTA, and 5% glycerol added with a phosphatase and protease inhibitor mixture (Roche Applied Sciences). Sepharose beads slurry was washed three times with the lysis buffer by centrifuging at 3000*g* for 2 min at 4°C. Then, the conjugated anti-FLAG antibody was carried out by overnight incubating the suspended Protein G Sepharose and anti-Flag monoclonal antibody (Sigma-Aldrich, F1804) at 4°C with continuous mixing. After washing three times with a lysis buffer, the mixture was incubated with the lysates at 4°C overnight with gentle mixing on a suitable shaker. Next, the precipitated protein with the bead was washed three times and analyzed using the immunoblotting technique as previously described (*76*). Whole-cell lysate has been used for the input or positive control. Anti-FLAG pull-down was performed for FLAG nonexpressing BCa cell line for the negative control. NPEPPS and LRRC8A have been probed using the rabbit polyclonal NPEPPS antibody (1:1000; Origene, TA308014), rabbit IgG polyclonal *LRRC8A* antibody (1:1000, LSBio, LS-C290818 and LS-B16989), and rabbit IgG polyclonal LRRC8D antibody (1:1000, Sino Biological, 104245-T32). For fractionated membranal/cytosolic Western blots, protein lysates were prepared using the Mem-PER Plus Membrane Protein Extraction Kit (Thermo Fisher Scientific) following the manufacturer’s guidelines and quantified using the standard BCA method. Co-IP sample preparation was performed similarly to that of the total protein lysates.

IP of unmodified (non–FLAG-tagged) cell lines was carried out with whole-cell lysates prepared from cultured cells and treated with 10% paraformaldehyde for 10 min before protein lysis using a RIPA lysis and extraction buffer (Thermo Fisher Scientific). All lysates were prepared on ice without adding Halt protease, phosphatase inhibitor cocktail, or EDTA. The protein concentration of lysates was quantified with a BCA protein assay (Pierce, Thermo Fisher Scientific). IP was performed according to the manufacturer’s instructions (Pierce Classic Magnetic IP/Co-IP Kit) with primary antibodies against NPEPPS (Invitrogen, PA5-83788), LRRC8A (LSBio, LS-C290818 and LS-B16989), and anti-mouse IgG (Sigma-Aldrich, A9044; MP Biomedicals, 855689). Western blot analysis was performed using whole-cell lysates and IP elution products as described above.

### siRNA-mediated knockdown experiments

*NPEPPS* (L-005979-00-0020), *LRRC8A* (L-026211-01-0020), *LRRC8B* (L-014003-00-0005), *LRRC8C* (L-017159-01-0005), *LRRC8D* (L-015747-01-0020), *LRRC8E* (L-016488-01-0005), and nontargeting (D-001810-10-20) siRNA SMARTpools were purchased from Horizon Discovery and resuspended in a Dharmacon 5X siRNA Buffer and then diluted to 1x with PBS. Transfections were performed using Lipofectamine RNAiMax (Thermo Fisher Scientific) transfection reagent according to the manufacturer’s specifications. Briefly, cells were grown to ~60% confluence in 6-well or 10-cm plates before being transfected and allowed to incubate overnight. The following day, cells were trypsinized and replated into 96-well plates at 1000 to 2000 cells per well and allowed to attach overnight. Cells from the initial transfection were also replated into 6-well plates to collect protein and RNA to confirm knockdown. The following day, cells were treated using their previously established resistance doses of gemcitabine, cisplatin, or GemCis (table S1), and their relative growth rates were measured on the IncuCyte ZOOM (Essen Bioscience) over time. For the CyTOF experiments, cells were grown in siRNA SMARTpools for 72 hours before beginning cisplatin treatment.

### shRNA-mediated knockdown experiments

The University of Colorado Cancer Center Functional Genomics Shared Resource carried out lentiviral production and transduction. Plasmids from The RNAi Consortium (TRC) collection (TRC construct numbers TRCN0000073838, TRCN0000073839, and TRCN0000073840) used for targeting *NPEPPS* were selected based on predicted knockdown efficiency; nontargeting controls used were SHC002 and SHC016. Two micrograms of the target shRNA construct and 2 μg of a 3:1 ratio of psPAX2 (Addgene) and pMD2.G (Addgene) were transfected into HEK293FT cells using 2 μg of polyethylenimine (Polysciences). A lentiviral particle–containing medium was filtered using a 0.45-μm cellulose acetate syringe filter and used for transduction. Puromycin selection was performed at doses used for CRISPR library screening, or in some cases, cells were reselected with higher doses of puromycin (10 μg/ml) to ensure complete elimination of nontransduced cells. Selected cells were frozen at early passage, and early passage cells were used for all experiments.

### CRISPR-Cas9–based gene editing for *NPEPPS* KO

*NPEPPS*-KO cell lines were generated from multiple cancer cell lines (KU1919, T24, 5637, Caov-3, and HEK293T) using parental and cisplatin-resistant derivatives. Cell lines were cultured as described above for at least 2 weeks before nucleofection and passaged 2 days before. Upon reaching 60 to 70% confluence, cells were washed 2x with warm PBS and gently dissociated with trypsin. The trypsin was neutralized by adding a 2x volume of warm cell culture media (described above). Cells were counted, and 1 × 10^6^ cells were transferred to a 15-ml conical tube and spun down at 90 RCF for 10 min at room temperature. After gently aspirating the supernatant, the cell pellet was carefully resuspended in 25 μl of the appropriate nucleofection solution (NFS) (described in detail below). The Cas9:pmaxGFP complex was prepared by combining 10.6 μl of NFS with 4 μl of the Cas9 enzyme (5 μM) and 2.4 μl guide RNA (gRNA) (1 μg/μl, from the Lonza Cell Line Optimization 4D-Nucleofector X Kit) in a sterile microcentrifuge tube and incubating at room temperature for 10 min. A total of 2 × 10^5^ cells (5 μl of cells in an NFS solution) were added to the corresponding Cas9:gRNA mixture, and the total volumes of each (20 μl) were transferred to separate wells on the nucleocuvette strip. Cells were electroporated using the appropriate programs of the Lonza 4D-Nucleofector X Unit (listed below). After nucleofection, cells were resuspended by adding 80 μl of the prewarmed cell culture medium to each sample well, and the total volumes were transferred to separate wells of a 96-well or 6-well plate and cultured as described above. After 24 to 48 hours of recovery, cells were expanded to 10- or 15-cm plates and collected for protein analysis by Western blot (as described above) to validate NPEPPS expression after editing.

#### NFSs and conditions

Optimal conditions were evaluated by transfecting the Cas9:pmaxGFP vector into target cells with each of the three provided NFS buffers (SE, SF, and SG) in the Lonza Cell Line Optimization 4D-Nucleofector X Kit and electroporating with each of the 16 preset programs on the Lonza 4D-Nucleofector X Unit. The following optimal conditions were chosen by green fluorescent protein (GFP) expression and cell viability. 5736-Par and 5637-Cis: SE buffer, program CM-150. KU1919-Par and KU1919-Cis: SG buffer, program DS-120. T24-Par and T24-Cis: SE buffer, T24-Par was electroporated with program CA-137 and T24-Cis with program DN-100. Caov-3-Par and Caov-3-Cis: SE buffer, program CM-150. HEK293T cells: SF buffer, program DS-150.

### Lentiviral transduction-based addback of *NPEPPS* variants to *NPEPPS*^−/−^ cells

As with shRNA-mediated knockdown, the University of Colorado Cancer Center Functional Genomics Shared Resource carried out lentiviral production and transduction for the NPEPPs addback cell lines. Custom NPEPPs FLAG-tagged overexpression plasmids were designed and then acquired from Vector Builder. These plasmids were pLV[Exp]-Bsd-CMV>hNPEPPs[NM_006310.4]/3xFLAG (NPEPPs-WT-OE), pLV[Exp]-Bsd-CMV>{hNPEPPs[NM_006310.4](E353V)}/3xFLAG (NPEPPs-mut-OE), and pLV[Exp]-Bsd-CMV>{Stuffer 300 bp} (EV). For packaging the plasmids into lentiviral particles, HEK293FT cells were seeded at 500,000 cells per well in 6-well plates on day 0. On day 1, viral packaging mix was prepared by combining 2.5 μg of the Vector Builder plasmid, 2 μg of a 2:1 ratio of psPAX2 (Addgene, plasmid #12260) and pMD2.G (Addgene, plasmid #12259), and 3 μg of polyethylenimine (Polysciences, catalog no. 23966) in 400 μl of Opti-MEM (Life Technologies, catalog no. 31985070). This plasmid mix was used to transfect HEK293FT cells. Media were changed 14 hours after the transfection on day 2 with fresh DMEM (Life Technologies, catalog no. 11965092) + 10% FBS (Gibco, catalog no. 16000044) + 1% antibiotic-antimycotic (Gibco, catalog no. 15240062). On day 4, the lentiviral particle–containing medium was filtered using a 0.45-μm cellulose acetate syringe filter and collected for subsequent transduction. For the transduction, 16 μg of polybrene was added to 2 ml of each lentiviral particle–containing medium. Then 500,000 T24-g82-KO cells were seeded in each well of a 6-well plate. Each well received either 2 ml of a lentiviral particle–containing medium or 2 ml of T24 complete growth media. The freshly seeded and transduced cells were centrifuged at 1000 rpm for 1 hour at room temperature. After centrifugation, the cells were stored in an incubator. Media were changed 20 hours after transducing the cells with fresh T24 complete growth media. The transduced T24-g82-KO cells were selected with blasticidin at a working concentration of 300 μg/ml in T24 complete growth media. During the selection, cells were split as necessary, and media were changed every 48 hours with fresh T24 complete growth media containing blasticidin. After 1 week under blasticidin selection, the nontransduced T24-g82-KO cells were observed to be completely dead, and the selection was complete.

### Intracellular cisplatin measurements using CyTOF

Cell lines were cultured for several passages in IMDM + 10% FBS (DMEM + 10% FBS for HEK293T). Before the experiment, cells were cultured in complete media to be 50 to 80% confluence overnight and then treated the next day with varying concentrations of cisplatin, carboplatin, or PBS as indicated and then dissociated after 4 hours of treatment. For dissociation, cells were washed twice with room temperature PBS and then incubated with PBS + 0.05% trypsin-EDTA for 10 to 15 min. Cells were neutralized with complete media and then fully dissociated into single-cell suspension by gentle pipetting. After dissociation, cells were counted by Trypan blue staining and then placed in separate tubes at 1 × 10^6^ cells. Individual samples were then fixed, permeabilized, and labeled using unique barcodes using the Cell-ID 20-plex Pd Barcoding kit (Fluidigm) according to the manufacturer’s protocol. Barcoded samples were pooled across cell line conditions and cisplatin concentration, incubated with Cell-ID Intercalator-Ir, mixed with equilibration beads, and acquired on a Helios mass cytometer (Fluidigm). Postacquisition data were normalized to equilibration beads and debarcoded, using the bead-normalization and single-cell-debarcoder packages from the Nolan Laboratory GitHub page. Relative cisplatin intensity (defined by ^195^Platinum isotopic mass intensity) was analyzed among nucleated ^191^Iridium+ ^193^Iridium+ events defined by Boolean gating within FlowJo (v10.7.1).

### Fluorescence microscopy and FRET-AB

T24 and KU1919 cells were cultured according to the cell culture methods described above. Cells were transduced for LRRC8A-FLAG, LRRC8A-mCherry, or NPEPPS-FLAG epitope tagging according to the lentiviral transduction method followed by antibiotic selection. Then, cells were cultured for three to five passages for proper recovery from antibiotic stress. For performing microscopy, cells (5 × 10^3^ to 10 × 10^3^ cells/ml) were seeded on 35-mm glass-bottom imaging dishes (μ-Dish 35 mm, high Glass Bottom; ibidi) with IMDM complete media. After harvesting overnight, the cells were washed with PBS and fixed with formaldehyde (3.7% in PBS, 15 min). After washing with PBS, cells were permeabilized with Tween 20 (0.2% in PBS; v/v) instead of membrane-damaging Triton-X–based detergent. Tween-20 is a nonionic detergent that creates pores on the membrane for antibodies to go through without dissolving the membrane (*77*). Hence, to keep the integrity of VRAC subunits on the plasma membrane, we adopted Tween 20–based permeabilization. After a 15-min incubation with Tween 20 (0.2% in PBS), cells were washed and treated with a blocking solution [1% BSA, glycine (22.52 mg/ml) in PBST (PBS + 0.1% Tween 20)] for 1 hour at room temperature. Then, cells were stained for either single color or dual by incubating overnight with mouse anti-FLAG (MilliporeSigma) or antibody cocktail of mouse anti-FLAG and rabbit anti-NPEPPS (Origene), respectively. For single staining, both LRRC8A-FLAG and NPEPPS-FLAG cells were stained with anti-FLAG. For dual staining of LRRC8A-FLAG cells or LRRC8A-mCherry, NPEPPS has been stained with its native antibody. After being washed with PBS (3X, 5 min each), cells were incubated with donkey anti-mouse IgG (H+L) Alexa Fluor 594 (Thermo Fisher Scientific) for single staining or with donkey anti-mouse IgG (H+L) Alexa Fluor 594 and donkey anti-rabbit IgG (H+L) Alexa Fluor 647 (Thermo Fisher Scientific) for dual staining. After incubation for 2 hours at room temperature followed by washing with PBS (3X, 5 min each) cells were observed under a Leica Stellaris 8-STED super-resolution confocal microscope using a 63X oil impulsion objective.

For mutant NPEPPS distribution, cells were stained with primary anti-FLAG antibody and secondary donkey anti-mouse IgG (H+L) and counterstained with a CellMask plasma membrane stain (Thermo Fisher Scientific) and Hoechst (33342) as a nuclear stain. Cells were stained for a microscopy-based FRET study using the dual staining method mentioned earlier. The study was conducted using a Stellaris 8-STED super-resolution confocal microscope with a 63X oil immersion objective. Here, we adopted the FRET-AB method to measure the FRET. Following the AB method (*78*), the measurement of donor fluorescence was performed both before (FRET condition) and after photobleaching the acceptor (non-FRET condition). By principle, energy transfer from the donor to the acceptor results in a reduction of donor fluorescence and an elevation of acceptor fluorescence. The presence of FRET between the donor and acceptor becomes apparent through the restoration of donor fluorescence subsequent to the targeted photobleaching of the acceptor population. The energy transfer efficiency is computed from the measured donor fluorescence intensity, both before and after AB, using the equation *E* = (*I*_d_ − *I*_da_)/*I*_d_, where *I*_da_ signifies the intensity of donor fluorescence in the acceptor’s presence (prebleach), and *I*_d_ represents the donor fluorescence intensity without the acceptor (postbleach). Statistical significance of the differences in energy transfer efficiency between groups was evaluated by the Mann-Whitney *U* test.

### Colocalization quantification by PCC

ImageJ and LAS X were used to analyze the confocal images. For the quantification of colocalization, the JACoP plugin was used (*79*–*81*). Costes’ automatic threshold was applied to obtain the overall PCC. Van Steensel’s cross-correlation functions were evaluated to observe the true value of the colocalization coefficient and to obtain δ*x* (pixel shift). In all analyses, perfect bell-shaped structures were represented with δ*x* ~ 0. The Colocalization Finder plugin was used to identify the location of colocalization in each cell. A threshold of PCC > 0.8 was considered to analyze the colocalization location. Scatterplots were used to adjust the cutoff, and the location of colocalization was visualized by the white spot generated by the plugin. This tool was also used to measure the PCC at different intracellular-to-membranal colocalizations of *NPEPPS* and *LRRC8A* in *LRRC8A*-mCherry transduced cells. Statistical significance was evaluated by unpaired *t* tests.

### Xenograft model

Six-week-old NU/J mice female mice were purchased from the Jackson Laboratory (J:NU/007850/homozygous for Foxn1) and kept in a specific pathogen–free environment at the Cedar-Sinai Medical Center animal facility. Mice were allowed to acclimatize for 1 week before starting xenograft. All the experiments were conducted based on the protocol approved by the Institutional Animal Care and Use Committee (IACUC) at the Cedar-Sinai Medical Center animal facility (study approval number IACUC008253, approved 15 January 2024).

For xenografts, mice were injected with 4 × 10^6^ KU1919-Cis cell lines [with NPEPPS^−/−(EV)^, NPEPPS^−/−(WT)^, or NPEPPS^−/−(E353V)^] in phenol red–free and serum-free RPMI, mixed with an equal volume of the Matrigel matrix to a total volume of 100 μl. After tumors were engrafted and reached the size of ~100 mm^3^, mice from each of the three cell line groups were randomized to either Dulbecco’s phosphate buffered saline (DPBS, Gibco) as a control or cisplatin (Sigma-Aldrich) treatment. The dilution and preparation of cisplatin were conducted as described before (*12*). Briefly, mice were treated three times weekly with cisplatin (2 mg/kg) via intraperitoneal injection, whereas controls were injected with an equal volume of DPBS. Tumor length and width were measured using calipers, and tumor volume was calculated using (*L* × *W*^2^)/2, where *L* is the largest diameter, and *W* is the width with the shortest perpendicular tumor measurement. Mouse health was accessed daily, and the endpoint was determined and performed following previously described guidelines (*12*). Tumor growth differences were evaluated by a two-way ANOVA mixed effects model, and *P* values for the differences in growth by genotype were reported per group. Weights of tumors and other organs (liver, kidney, and spleen) were accessed after the endpoint of each mouse. Statistical analyses of tumor or organ weights were completed with one-way ANOVA test comparing the mean of each group to the mean of the control group.

### Flow cytometry analysis and quantification of FLAG and assessment of DNA double-strand break

Each tumor sample was mechanically disrupted after the endpoint and passed through a 70-μm filter to make single cells. Then, cells were washed and incubated with Fc block (anti-mouse CD16/CD32 monoclonal antibody) to avoid Fc-mediated binding of antibodies. Surface staining has been conducted using HLA-ABC-PE (Invitrogen) or iso-PE (BioLegend). Live cells were accessed by the L/D ghost dye (UV 450) (Cell Signaling Technology) staining. Then, cells were fixed and permeabilized (BD Biosciences) for intracellular staining with anti-FLAG-AF488 and anti-Phospho-Histone H2A.X (Ser^139^) (HisH2AXS139-1B3)–APC (Invitrogen). Isotypes ISO-AF488 (BioLegend) and ISO-APC (BioLegend) have been used as a control for staining. The four-color compensation has been performed for acquisition using a BD Biosciences Symphony A5 Cell Analyzer. Then, cells with HLA-ABC and FLAG-positive cells were gated to observe FLAG-positive KU1919 cells and these cells are further sorted for the NPEPPS-FLAG Phospho-Histone H2A.X (Ser^139^)–APC mean fluorescence intensity (MFI) to quantify the double-strand breaks (DSBs) in the samples. Gating for HLA-ABC, FLAG-NPEPPS, and DSBs is provided in the Supplementary Materials.

### Enzymatic assays (in vitro and ex vivo)

Protein lysates were prepared using the previously mentioned IP lysis buffer. The lysates underwent buffer exchange with PBS using an Amicon U0000ltra-0.5 centrifugal filter unit (3000-Da molecular weight cutoff Ultracel membrane; MilliporeSigma). Subsequently, the protein concentration was determined, and the protein was adjusted to a final concentration of ~2 μg/μl. Next, 10 μg of the protein was loaded onto a polystyrene black assay plate (with light blocking flat-bottom, for fluorescence assays; Stellar Scientific) containing an assay buffer [25 mM Hepes and 1 mM DTT (pH 7.0)], except for the blank. The reaction was initiated by adding 10 μM of the substrate Leu-AMC (H-Leu-AMC; Bachem) to the wells, including the blank. The catalytic activity was assessed by measuring the fluorescence at excitation and emission wavelengths of 380 and 460 nm, respectively, using a kinetic mode on the BioTek Synergy H1 plate reader.

### Survival analyses

Seiler *et al.* described a cohort of 343 patients with muscle-invasive BCa (*43*). Similarly, Yoshihara *et al.* described a cohort of 110 patients with HGSC (*51*). In each case, the authors provided the GE microarray data and clinical metadata for patients in the cohort. Patients with a record of platinum chemotherapy treatment and survival between 0 and 61 months (*n* = 223 for BCa; *n* = 76 for HGSC) were stratified based on mRNA up-regulation (above/below median) of target proteins as labeled. The log-rank test was used to test the difference in overall survival between the stratified patient groups. Cox proportional hazard ratios were generated using the coxph command in the Survival package in R (version 4.3.0).

GE, survival data, and treatment information for patients with cervical squamous cell cancer in the TCGA cohort (PanCancer Atlas) were downloaded from cBioPortal (*59*, *61*). Patients were included if they had a record of platinum-based treatment and survival between 0 and 61 months to remove outliers from the dataset (*n* = 92) and then stratified based on mRNA expression of target proteins as labeled. The log-rank test was used to test the difference in overall survival between the stratified patient groups. Cox proportional hazard ratios were generated using the coxph command in the Survival package in R (version 4.3.0).

### Patient-derived organoid models

Patient-derived BCa organoid cultures were established as previously described (*12*). Briefly, sequencing libraries were prepared using the 3′ mRNA-Seq Library Prep Kit Protocol for Ion Torrent (QuantSeq-LEXOGEN, Vienna, Austria), with sequencing performed on an Ion Proton System. Bam files for each RNA-seq sample were summarized to a 3′ untranslated region read counts table using the Bioconductor R package GenomicRanges (*82*), and read counts were normalized using DESeq2 (*83*).

Ex vivo cisplatin response data were either repurposed (patients 2 to 4) (*12*) or generated for this study (patients 1 and 5) using the same methods as previously described. We refer to Jones *et al.* (*12*) for a detailed description of experimental procedures. Briefly, tumoroids were treated with cisplatin ranging from 0.1 to 40 μM for 6 days, followed by viability assessment using CellTiter-Glo 3D (Promega, #G9681). Viability data were normalized using tumoroid wells treated with a vehicle control. IC_50_ values were estimated with GraphPad Prism (version 9.3.1) using a variable slope, four-parameter nonlinear regression model.
