# Supplementary material for: Regulation of volume-regulated anion channels alters sensitivity to platinum chemotherapy
Source: Sci Adv. 2024 Dec 13;10(50):eadr9364. doi: 10.1126/sciadv.adr9364 (PMC11641020; doi:10.1126/sciadv.adr9364)
Supplement: Supplementary file 1 — Figs. S1 to S15 Legends for tables S1 to S3 [file sciadv.adr9364_sm.pdf]

Science Advances
AAAS

Supplementary Materials for

Regulation of volume-regulated anion channels alters sensitivity to platinum chemotherapy

Lily Elizabeth R. Feldman *et al.*

Corresponding author: Dan Theodorescu, dan.theodorescu@cshs.org;
James C. Costello, james.costello@cuanschutz.edu

Sci. Adv. **10**, eadr9364 (2024)
DOI: 10.1126/sciadv.adr9364

The PDF file includes:

Figs. S1 to S15
Legends for tables S1 to S3

Other Supplementary Material for this manuscript includes the following:

Tables S1 to S3

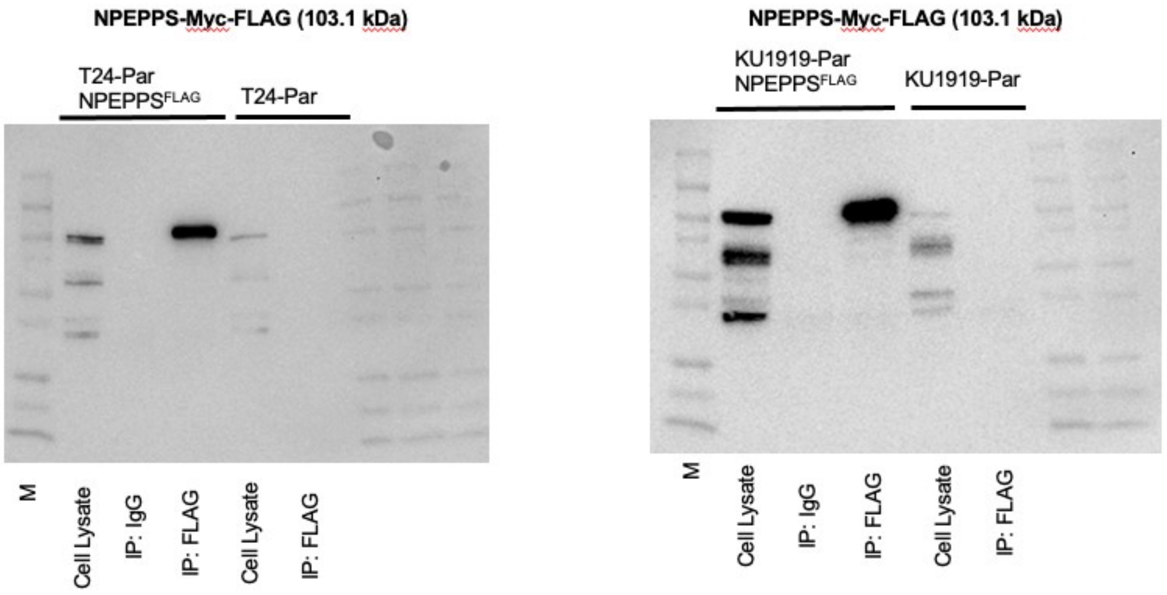

Figure S1. Validation of NPEPPS^{FLAG} cell lines used in Immunoprecipitation-Mass Spectrometry. Western blots of anti-NPEPPS primary antibody following immunoprecipitation with anti-IgG (negative control) or anti-FLAG primary antibody to validate the expression of NPEPPS^{FLAG} in T24-NPEPPS^{FLAG} (left) and KU1919-NPEPPS^{FLAG} (right) cell lines used in the IP-LC-MS/MS shown in **Figure 1**.

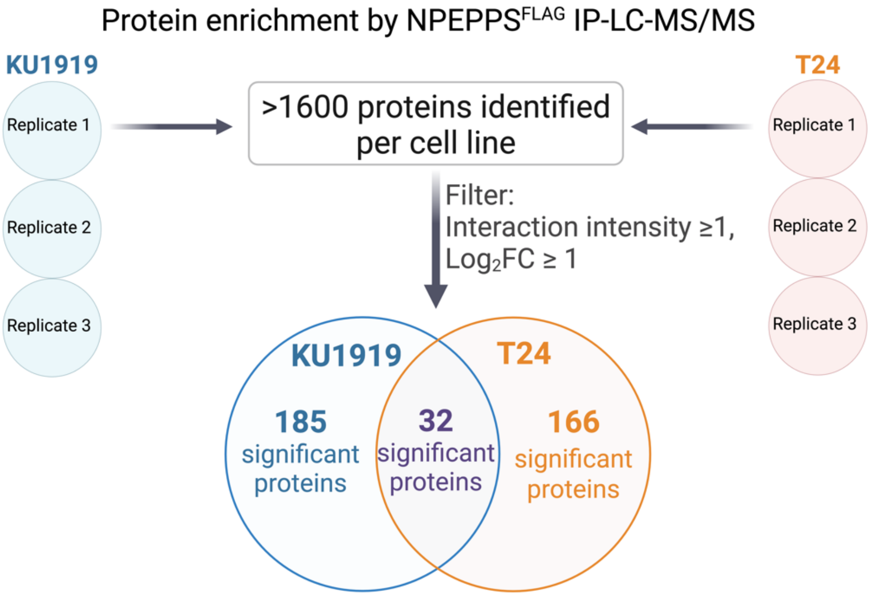

Figure S2. Schematic of the filtering steps resulting in 32 top interactor hits from NPEPPS^{FLAG} interactome. Immunoprecipitation-tandem Mass Spectrometry (IP-MS/MS) was conducted in biological triplicate for each cell line (T24-NPEPPS^{FLAG} and KU1919-NPEPPS^{FLAG}), identifying over 1600 protein interactors per cell line. These results were filtered for median interaction intensity greater than one and log_2FC greater than one compared to control (isotype) immunoprecipitation, resulting in 185 proteins considered significant in all KU1919 replicates and 166 in all T24 replicates. Finally, the protein interactors commonly significant across all KU1919 and T24 replicates were taken as the top interactors, a list of 32 proteins shown in **Figure 1**. Figure created using Biorender.com.

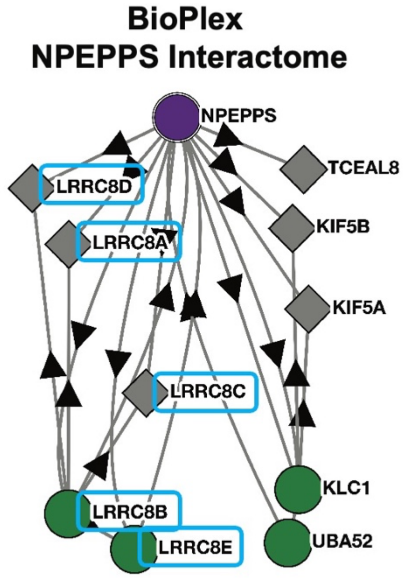

Figure S3. The NPEPPS interaction partners reported by the BioPlex Interactome. The BioPlex interactome (*35*) is a published database of protein interactions. Among the identified NPEPPS interactors, all five subunits of the Volume Regulated Anion Channel are present (LRRC8A, LRRC8B, LRRC8C, LRRC8D, and LRRC8E, highlighted in blue).

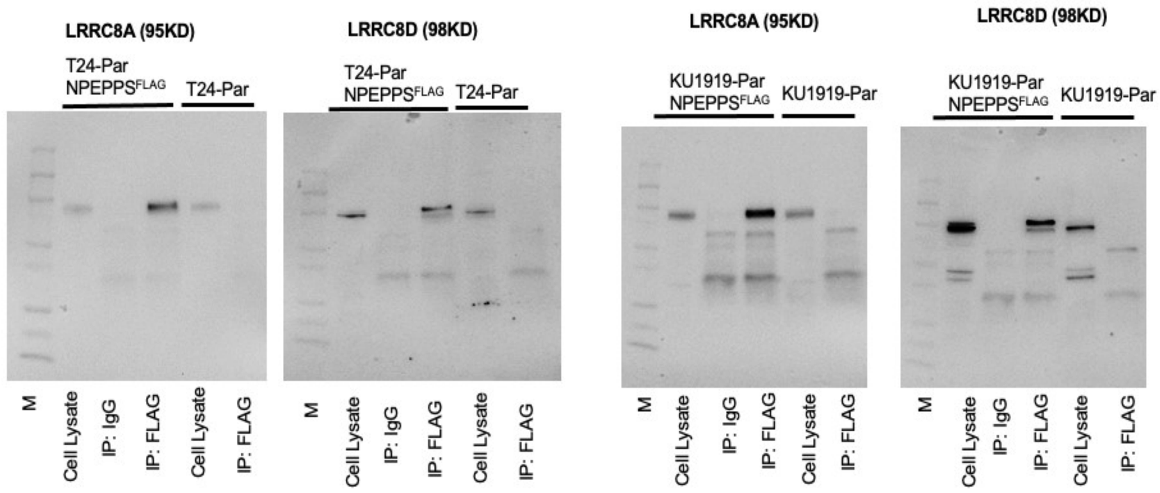

Figure S4. Validation of NPEPPS^{FLAG} cell line interactions. Western blots with anti-LRRC8A and anti-LRRC8D primary antibodies following immunoprecipitation with anti-IgG (negative control) or anti-FLAG primary antibody to validate the interactomes of NPEPPS^{FLAG} in T24-NPEPPS^{FLAG} (left) and KU1919-NPEPPS^{FLAG} (right) cell lines used in the IP-MS/MS shown in **Figure 1**.

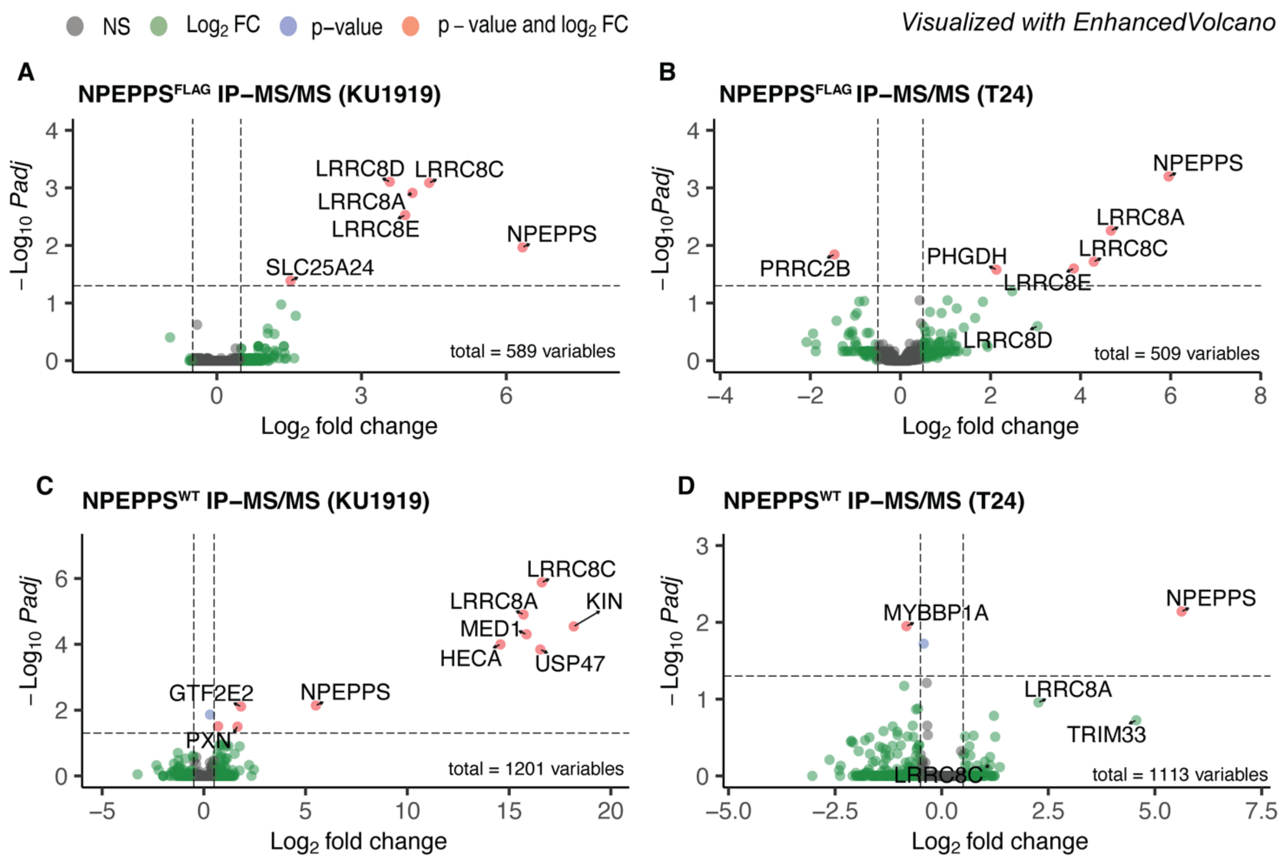

Figure S5. Volcano plots showing interactomes of NPEPPS^{FLAG} and NPEPPS^{WT} (native). Interactomes of (**A, C**) KU1919 and (**B, D**) T24 cells with native NPEPPS or overexpression of NPEPPS^{FLAG} were collected by tandem Mass Spectrometry following immunoprecipitation with either anti-FLAG or anti-NPEPPS primary antibody. Results were generated using the EnhancedVolcano package in R. Points are labeled as follows: NS (grey) = not significant, Log$_2$FC (green) = Log$_2$FC greater than one, p-value (blue) = adjusted p-value below 0.05, p-value and Log$_2$FC (red) = Log$_2$FC greater than one and adjusted p-value below 0.05.

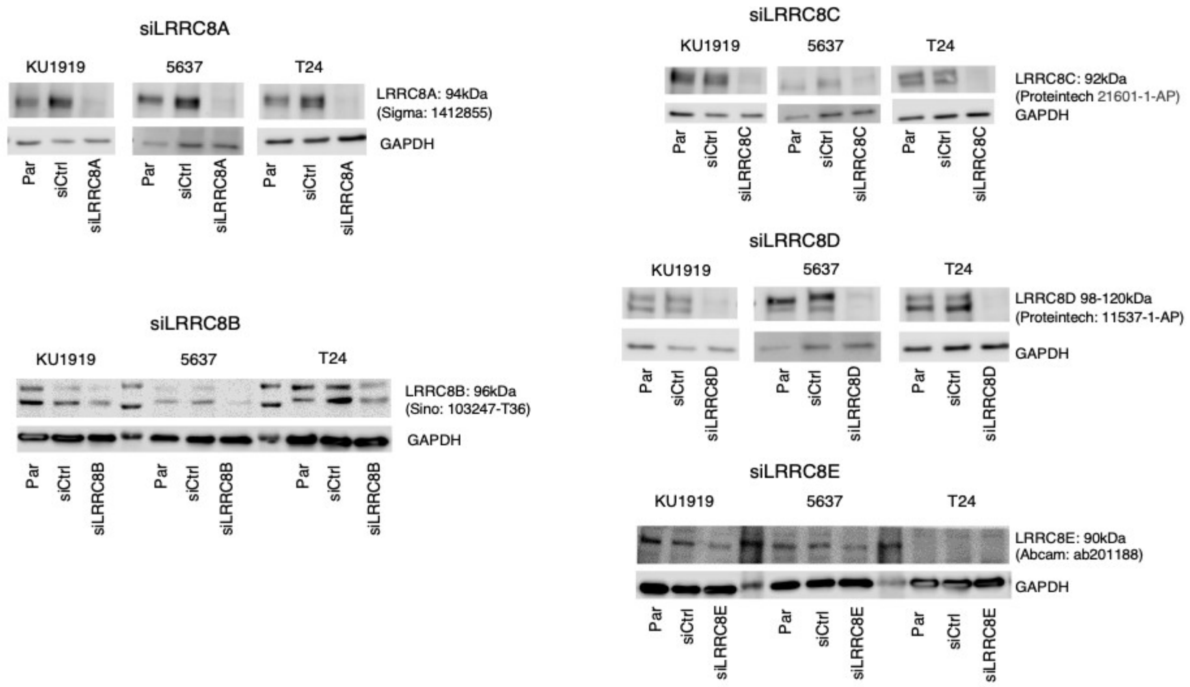

Figure S6. Validation of VRAC subunit knockdown following siRNA treatment before CyTOF analysis. Western blots of LRRC8A-E after 72 hours of siRNA treatment in KU1919, 5637, and T24 cells. Protein lysates shown here were collected within 48 hours of CyTOF analysis.

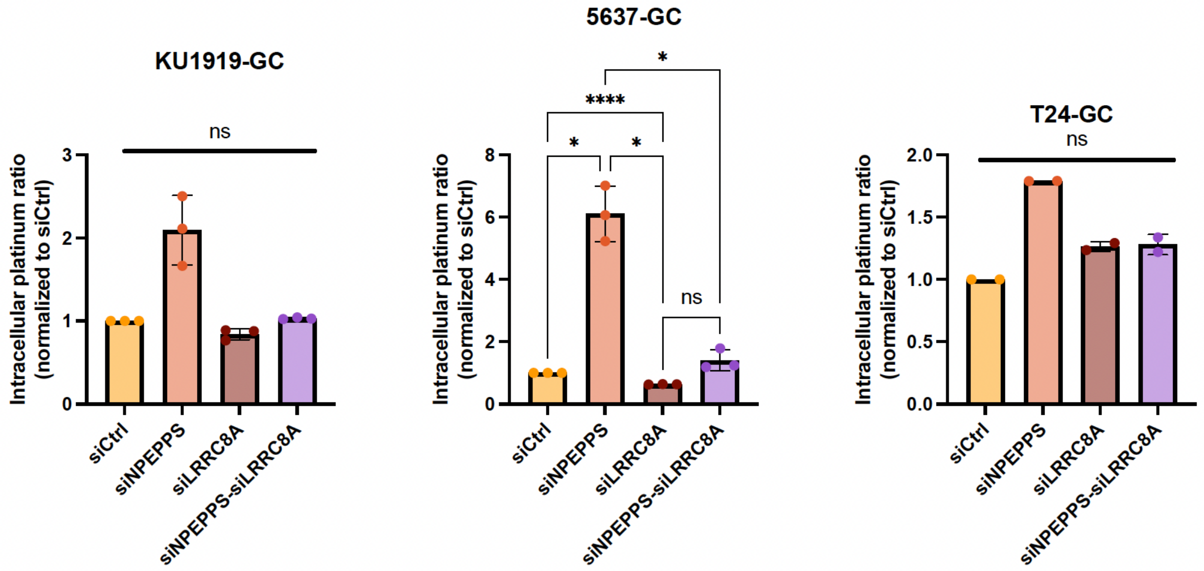

Figure S7. Normalized CyTOF results from GemCis-resistant cells. CyTOF in GemCis-resistant KU1919, 5637, and T24 cells shows intracellular cisplatin levels after 4 hours of 10 µM cisplatin with siRNA-mediated suppression of VRAC subunits *LRRC8A-E* compared to control (scramble) siRNA. Median intracellular cisplatin measurements across biological triplicates were normalized to the siRNA control and compared using a one-way ANOVA (**p<0.01, ***p<0.001).

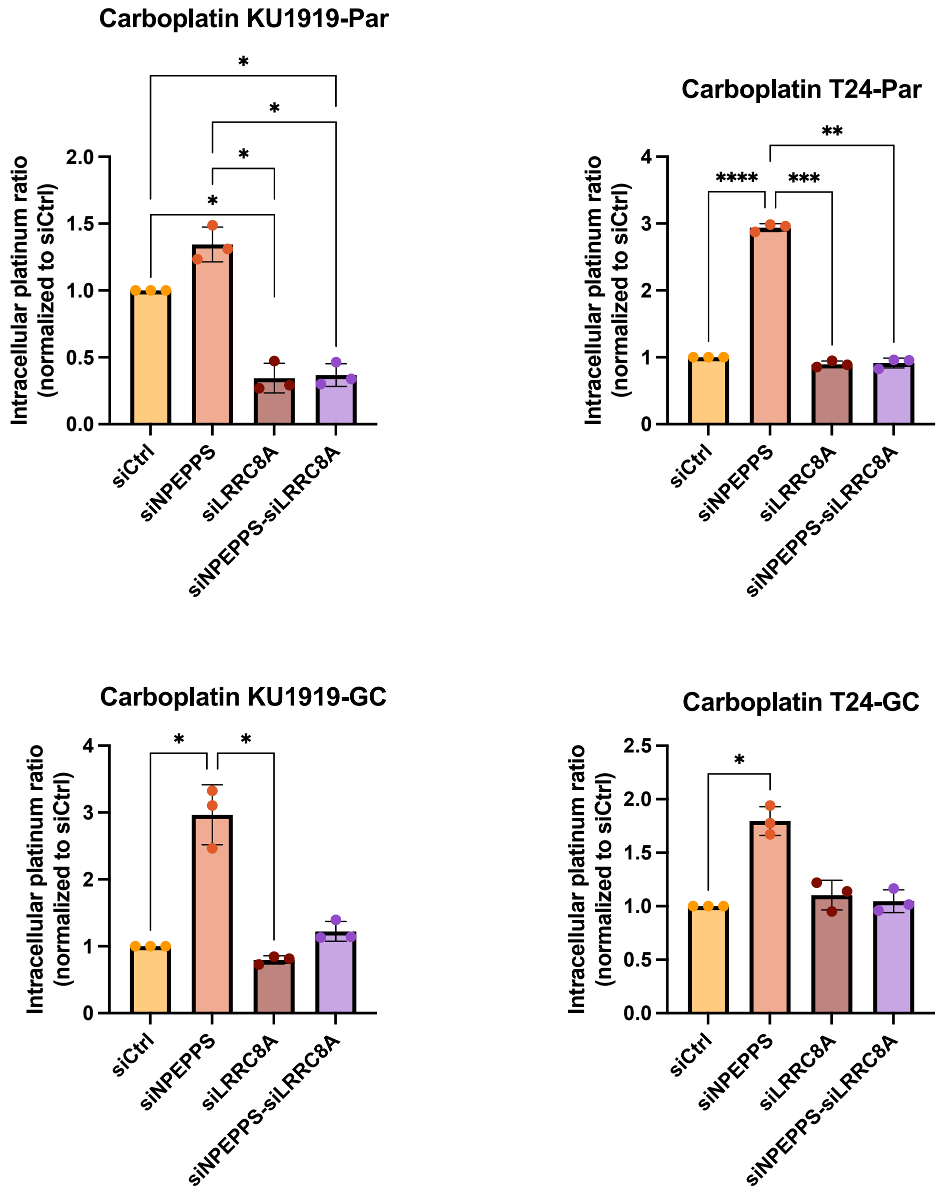

Figure S8. Normalized CyTOF results from sensitive Parental or GemCis-resistant cells following carboplatin treatment. CyTOF in Parental or GemCis-resistant KU1919, 5637, and T24 cells shows intracellular cisplatin levels after 4 hours of 100 μM carboplatin with siRNA-mediated suppression of VRAC subunits LRRC8A-E compared to control (scramble) siRNA. Median intracellular cisplatin measurements across biological triplicates were normalized to the siRNA control and compared using a one-way ANOVA (*$p<0.05$, **$p<0.01$, ***$p<0.001$).

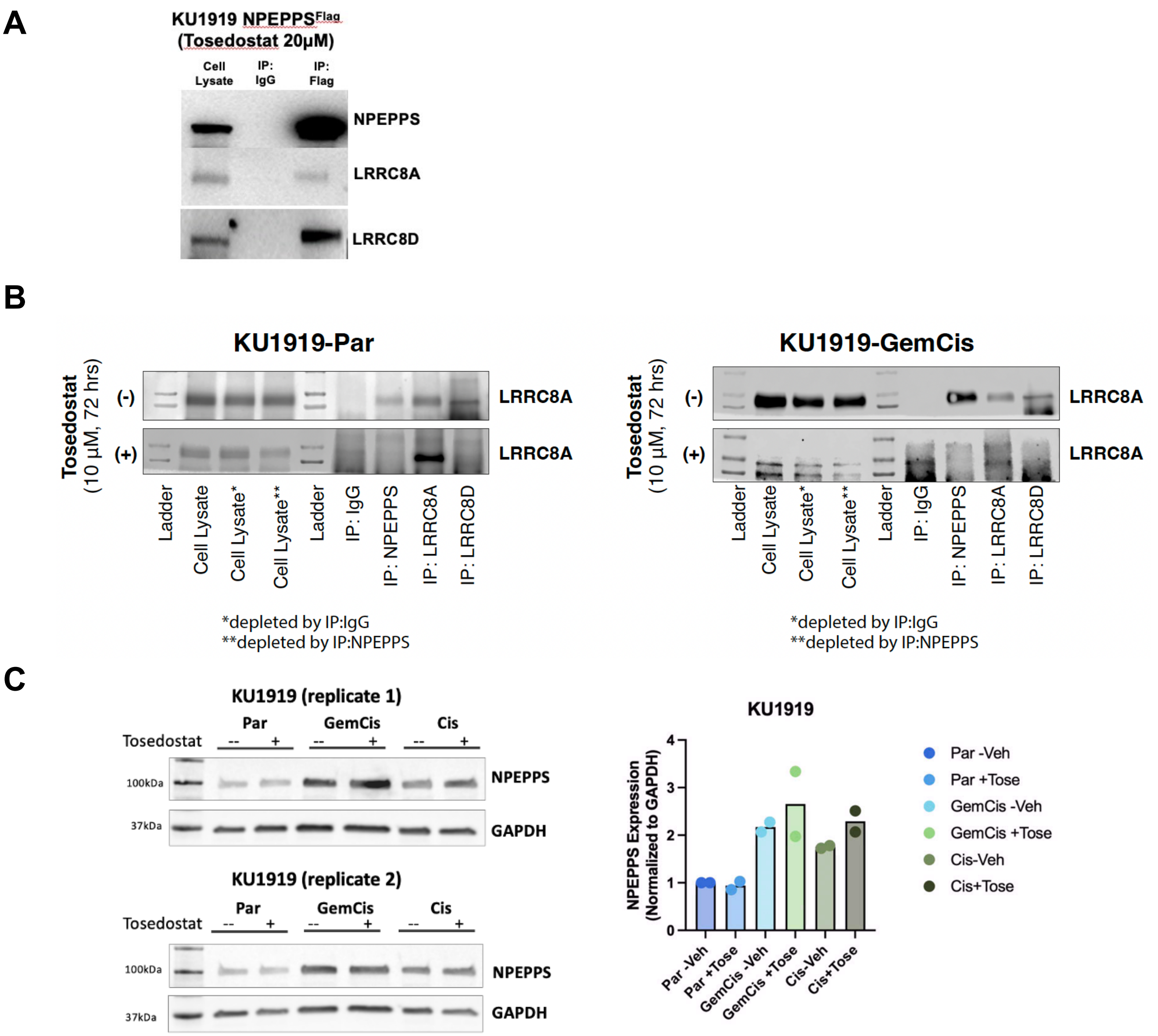

Figure S9. NPEPPS and LRRC8A/D changes after tosedostat treatment. (**A**) IP-Western blot analysis of NPEPPS^{FLAG} cells with tosedostat treatment (20 µM). Western blots with NPEPPS, LRRC8A, and LRRC8D primary antibodies following immunoprecipitation with anti-IgG (negative control) or anti-FLAG primary antibody to validate the interactomes of NPEPPS^{FLAG} in KU1919- NPEPPS^{FLAG} cell lines. Western blots for NPEPPS and LRRC8A following anti-FLAG or anti-IgG immunoprecipitation without tosedostat treatment are shown in **Supplementary Figures S1** and **S4**, respectively. (**B**) IP-Western blot analysis of KU1919 Par and GemCis cells with and without tosedostat treatment (10 µM). Western blots with anti-LRRC8A primary antibodies following immunoprecipitation with anti-IgG (negative control), anti-NPEPPS, anti-LRRC8A, or anti-LRRC8D primary antibodies. (**C**) Western blot analysis of NPEPPS expression in KU1919 Par, GemCis, and Cis cells with tosedostat treatment (10 µM) or vehicle. Western blots with anti-NPEPPS and anti-GAPDH primary antibodies from Parental, Cis-, or GemCis-resistant KU1919 (left) cells treated with tosedostat (10 µM in DMSO) or vehicle (0.1% DMSO) for 72 hours. Biological replicates are shown.

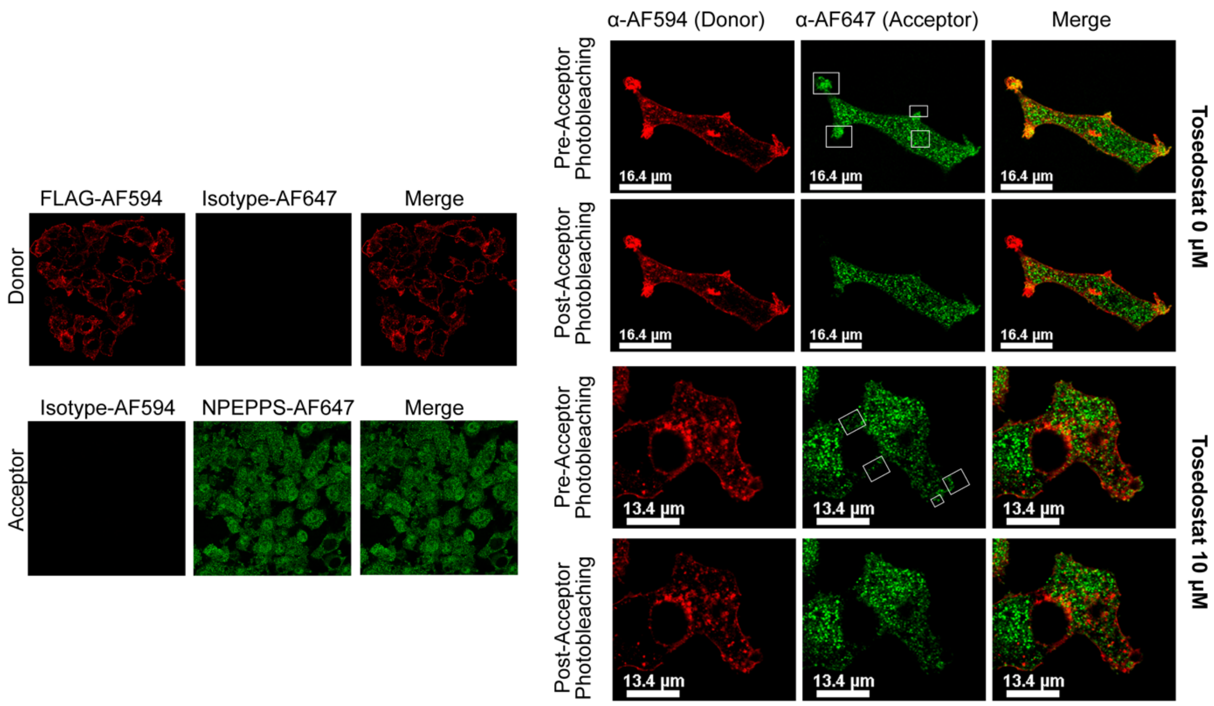

Figure S10. FRET-Acceptor-Bleaching images in cells with or without tosedostat treatment (10 µM, 24 hours). KU1919-LRRC8A^{FLAG} cells were stained with anti-FLAG-AlexaFluor594 (AF594, red) and anti-NPEPPS-AlexaFluor647 (AF647, green) for Förster Resonance Energy Transfer-Acceptor Bleaching (FRET-AB). Left: validation of the efficiency and specificity of staining with α-FLAG-AF594 and α-NPEPPS-AF647 shown with Isotype (IgG)-AF647 or -AF594 negative controls. Right: FRET-AB with or without tosedostat treatment (10 µM, 24 hours) before staining and imaging, shown before and after acceptor-photobleaching of AF647. Donor (AF594) images before and after acceptor-photobleaching are included as a control condition.

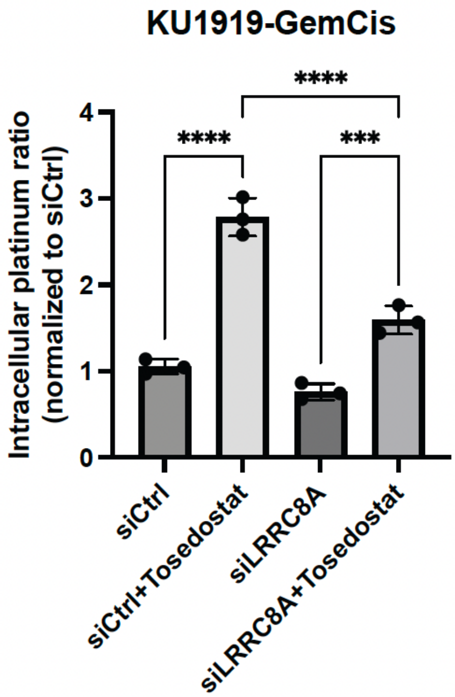

Figure S11. CyTOF of KU1919-GemCis cells with siLRRC8A and tosedostat treatment (20 µM, 24 hours). CyTOF in gemcitabine plus cisplatin-resistant KU1919 cells shows intracellular cisplatin levels after 4 hours of 10 µM cisplatin with siRNA-mediated suppression of VRAC subunit *LRRC8A* compared to control (scramble) siRNA, with or without tosedostat treatment (20 µM, 24 hours). Median intracellular cisplatin measurements across biological triplicates were normalized to the siRNA control without tosedostat treatment and compared using a one-way ANOVA (***p<0.001, ****p<0.0001).

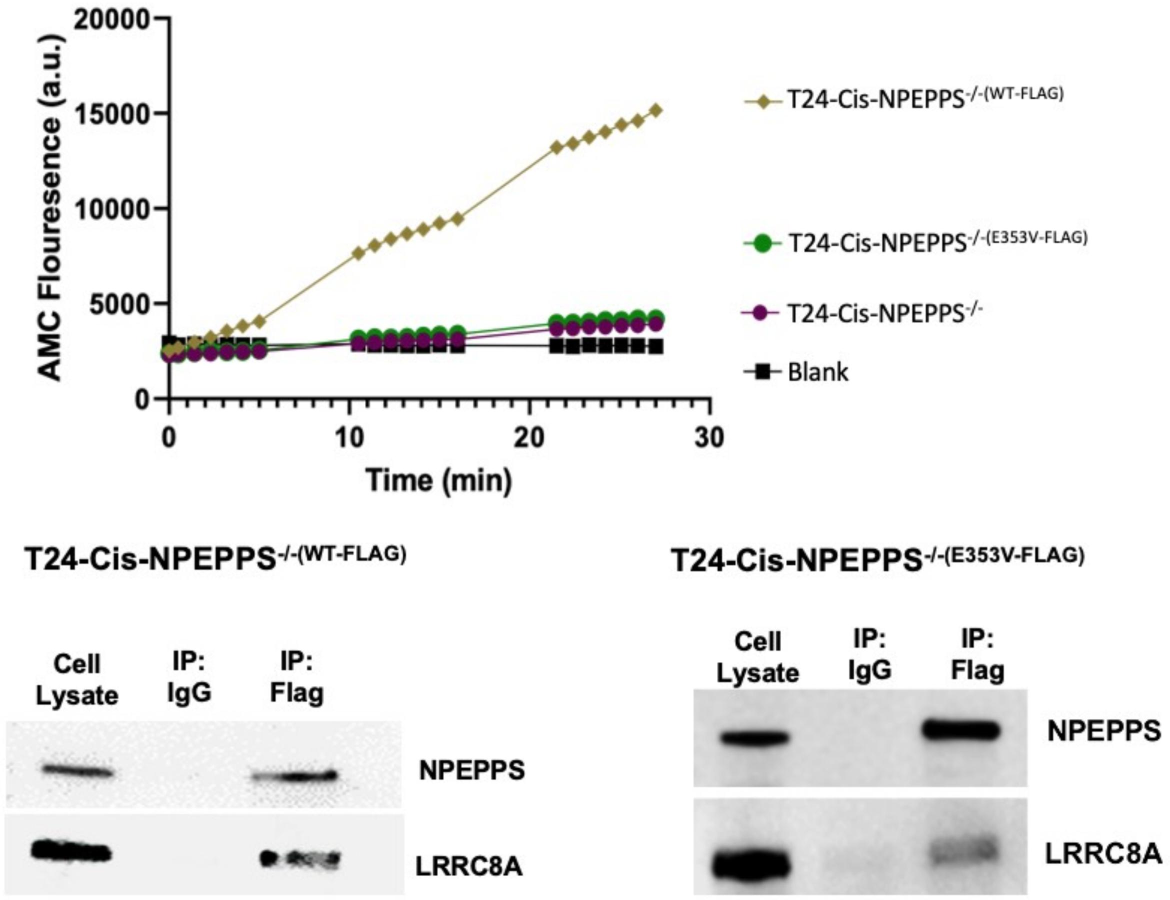

Figure S12. Validation of the enzymatic activity and binding patterns of cisplatin-resistant T24 cells with different versions of the NPEPPS protein. T24-Cis-NPEPPS$^{-/-}$ (NPEPPS-knockout) cells were engineered to express the following variations of NPEPPS: wild-type add-back (NPEPPS$^{-/-(WT\text{-}FLAG)}$) or catalytic-dead mutant add-back (NPEPPS$^{-/-(E353V\text{-}FLAG)}$). Enzymatic activity was measured with the fluorescent Leu-AMC reporter assay described in the Methods section. Western blots were performed with anti-NPEPPS, anti-LRRC8A, or anti-LRRC8D primary antibodies following immunoprecipitation with anti-FLAG or anti-IgG (negative control) antibodies.

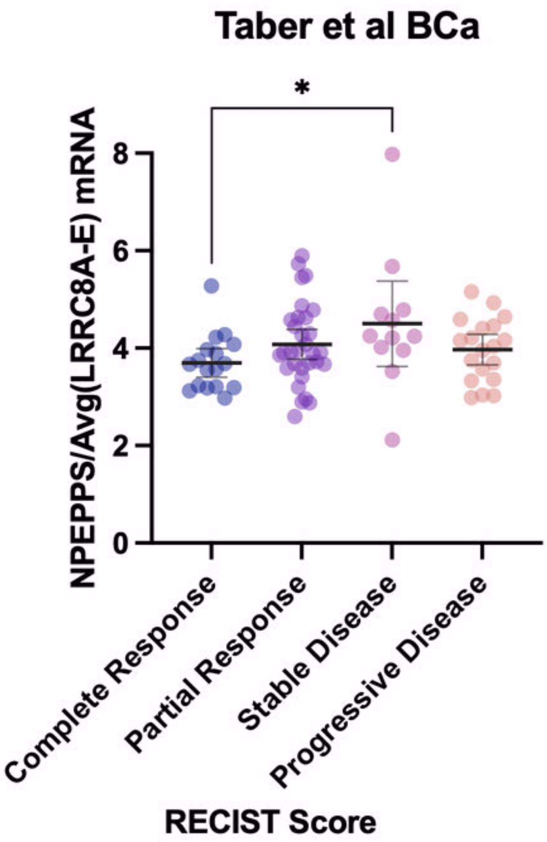

Figure S13. Gene expression and clinical response to platinum chemotherapy.
Normalized mRNA expression of *NPEPPS/*Avg*(LRRC8A-E)* in 79 BCa patients before treatment with platinum chemotherapy, grouped by clinical response per Response Evaluation Criteria in Solid Tumors (RECIST) scoring. Data originally reported in Taber et al. (*45*). *$p<0.05$ by One-Way ANOVA.

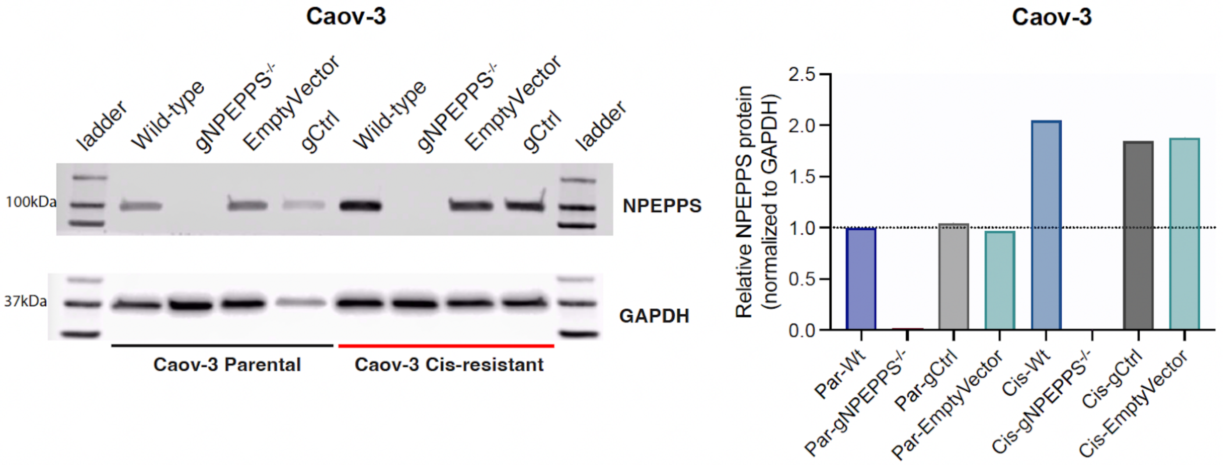

Figure S14. NPEPPS protein expression in Caov-3 cells with CRISPR/Cas9-mediated knockout of NPEPPS. Left: Western blots with anti-NPEPPS and anti-GAPDH primary antibodies in Caov-3 Parental and Cis-Resistant cells with wild-type NPEPPS or the following CRISPR/Cas9 gene editing conditions: gNPEPPS$^{-/-}$ (NPEPPS knockout), EmptyVector (no Cas9 enzyme) or gCtrl (control NonTargeting gRNA). Right: Densitometry of NPEPPS expression normalized to GAPDH expression showing upregulation of NPEPPS in Cis-resistant cells compared to Parental cells and efficient knockout of NPEPPS by CRISPR/Cas9-editing (gNPEPPS$^{-/-}$) in both Parental and Cis-resistant cells compared to gCtrl or EmptyVector control conditions.

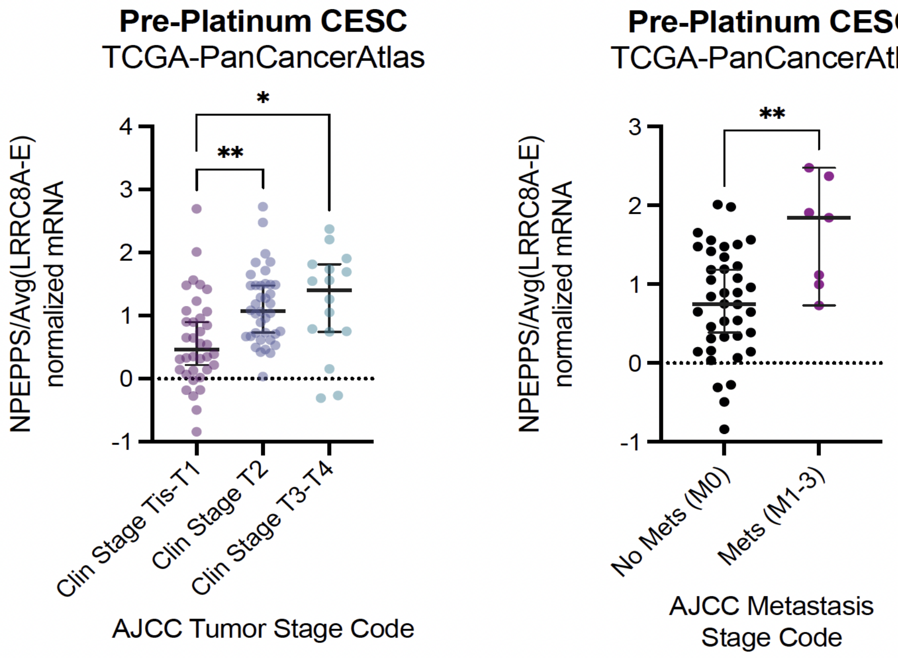

Figure S15. Correlation between *NPEPPS*/Avg*(LRRC8A-E)* expression and clinical measures in Cervical Cancer. Cervical Cancer (CESC) gene expression ratios from the TCGA-PanCancerAtlas study stratified by clinical stage (American Joint Committee on Cancer (AJCC) Tumor Stage Code) and by presence or absence of metastatic sites (AJCC Metastasis Stage Code). *p<0.05, **p<0.01 by One-Way ANOVA for clinical stage and **p<0.001 by unpaired t-test for metastasis stage.

SUPPLEMENTARY TABLE LEGENDS

Supplementary Table S1. Processed data from LC-MS/MS of anti-FLAG or anti-IgG (isotype control) immunoprecipitation complexes in KU1919 and T24 cells expressing $\mathrm{NPEPPS}^{\mathrm{FLAG}}$.

Supplementary Table S2. Processed data from untargeted metabolomics in KU1919 and T24 cells and targeted quantification of taurine in T24 cells under the tonicity and cisplatin treatment conditions described in Figure 2A.

Supplementary Table S3. Processed growth rate and growth inhibition data in response to a cisplatin dose course for 120 hours in KU1919-Cis cells with $\mathrm{NPEPPS}^{\mathrm{WT}}$, $\mathrm{NPEPPS}^{-/-(\mathrm{EV})}$, $\mathrm{NPEPPS}^{-/-(\mathrm{WT\text{-}FLAG})}$, or $\mathrm{NPEPPS}^{-/-(\mathrm{E353V\text{-}FLAG})}$.
